# The HSV-1 encoded CCCTC-binding factor, CTRL2, impacts the nature of viral chromatin during HSV-1 lytic infection

**DOI:** 10.1371/journal.ppat.1012621

**Published:** 2024-10-07

**Authors:** Pankaj Singh, Liqian Zhu, Mason A. Shipley, Ziyun A. Ye, Donna M. Neumann

**Affiliations:** 1 Department of Ophthalmology and Visual Sciences, University of Wisconsin- Madison, Madison, Wisconsin, United States of America; 2 College of Life sciences, Hebei University, Baoding, China; Cornell University, UNITED STATES OF AMERICA

## Abstract

HSV-1 genomes are rapidly heterochromatinized following entry by host cells to limit viral gene expression. Efficient HSV-1 genome replication requires mechanisms that de-repress chromatin associated with the viral genome. CCCTC-binding factors, or CTCF insulators play both silencing and activating roles in cellular transcriptional regulation. Importantly, the HSV-1 genome encodes several CTCF insulators that flank IE genes, implying that individual HSV-1 encoded CTCF insulators regulate IE transcription during all stages of the HSV-1 life cycle. We previously reported that the HSV-1 encoded CTCF insulator located downstream of the LAT (CTRL2) controlled IE gene silencing during latency. To further characterize the role of this insulator during the lytic infection we leveraged a ΔCTRL2 recombinant virus to show that there was a genome replication defect that stemmed from decreased IE gene expression in fibroblasts and epithelial cells at early times following initiation of infection. Further experiments indicated that the defect in gene expression resulted from chromatin inaccessibility in the absence of the insulator. To elucidate how chromatin accessibility was altered in the absence of the CTRL2 insulator, we showed that enrichment of Alpha-thalassemia/mental retardation, X-linked chromatin remodeler (ATRX), and the histone variant H3.3, both of which are known for their roles in maintaining repressive histone markers on the HSV-1 viral genome were increased on IE regions of HSV-1. Finally, both H3K27me3 and H3K9me3 repressive histone marks remained enriched by 4 hours post infection in the absence of the CTRL2 insulator, confirming that the CTRL2 insulator is required for de-repression of IE genes of viral genomes. To our knowledge these are the first data that show that a specific CTCF insulator in the HSV-1 genome (CTRL2) regulates chromatin accessibility during the lytic infection.

## Author summary

Herpes Simplex Virus 1 (HSV-1) remains a significant lifelong human pathogen. Over 70% of adults worldwide are infected with HSV-1 and following the establishment of a persistent latent infection in the peripheral nervous system, the virus can undergo reactivation. These reactivation episodes can result in recurrence of viral infection in the cornea and repeated episodes of recurrence can result in corneal blindness. HSV-1 remains a leading infectious cause of blindness worldwide. On a molecular level, both the incoming lytic virus and the latent viral genome are chromatinized for the purposes of silencing the virus. Viral gene expression in both lytic and reactivated HSV-1 requires the de-repression of chromatin on the viral genome but it remains unclear whether distinct transcriptional mechanisms regulate viral gene expression during these two stages of the viral life cycle. CCCTC-binding factors, also known as CTCF insulators, are essential regulators of chromatin structure and gene expression in mammalian cells and these cellular insulator elements also play vital regulatory roles in transcriptional control of all classes of herpesviruses as well. Existing research on CTCF and herpesviruses have been done in the context of herpesvirus latency but considering role of CTCF insulators in organizing chromatin architecture, it is logical that these important elements also play a role in either the initial chromatinization of virus, the de-repression of chromatin on incoming viruses so that transcription can occur- or both. Here we show that the CTRL2 insulator is required for de-repression of IE genes during the lytic infection and that the insulator has distinct and divergent functions during the lytic and latent stages of the viral lifecycle. To our knowledge these are the first data that show that a specific CTCF insulator in HSV-1 regulates chromatin accessibility in a site-specific manner. Understanding these mechanisms is particularly important for future drug discovery where cellular elements contributing to the de-repression of viral chromatin might be targeted.

## Introduction

Herpes Simplex Virus 1 (HSV-1) establishes a lifelong infection in the human host. During the primary infection, the virus infects epithelial cells and fibroblasts, where it undergoes genome replication, produces progeny virus and spreads by retrograde axonal transport to sensory neurons in the peripheral nervous system, establishing a persistent latent infection. Importantly, latent HSV-1 genomes can reactivate in response to external stimuli, leading to recurrent disease [1,2]. Following reactivation, HSV-1 travels by anterograde axonal transport to the periphery where it results in ocular pathogenesis that can lead to blindness [3]. Both the incoming lytic virus and the latent viral genome are chromatinized for the purposes of silencing the virus. Nonetheless, the mechanisms that govern the de-repression of chromatin on the viral genome in the initial lytic infection and mechanisms that dictate how HSV-1 exits latency and enters reactivation, where viral genes are ultimately de-repressed [4–6], are not fully understood and it remains unclear whether the virus utilizes distinct transcriptional mechanisms to regulate viral gene expression during these two stages of the viral life cycle.

Upon entry, the HSV-1 genome is rapidly heterochromatinized by the host cell by 1–2 hours post-infection [7–10]. This attempt at silencing the viral genome by the host cell is counteracted, in part, through chromatin destabilization by viral proteins VP16, ICP4 and ICP0. Both VP16 and ICP4 play a role in dysregulating nucleosome stability on incoming viral genomes, while ICP0 targets and degrades chromatin remodeling proteins needed for maintaining genome silencing [11–14]. Subsequently, VP16 colocalizes with the cellular protein Host Cell Factor-1 (HCF-1) in the cytoplasm and the complex enters the nucleus of the cell where it colocalizes with the cellular protein Octamer binding transcription factor 1 (Oct-1) bound to TAATGARAT sites on viral immediate early (IE) promoters and drives transcription [9]. This interaction facilitates the recruitment of histone demethylases that remove heterochromatic marks and methyltransferases that add euchromatic marks, leading to activation of transcription of viral genes in a complex and dynamic dance between the host cell and the virus as HSV-1 progresses through lytic genome replication [9,15–19]. In contrast, during latency, HSV-1 genomes are regularly chromatinized and stably associated with histones in sensory neurons, suggesting divergent mechanisms might control transcription during different stages of the virus life cycle. During latency, viral genomes maintain repressive histone marks, except for the Latency Associated Transcript (LAT), which maintains transcriptionally permissive histone marks [20–24]. For HSV-1 to reactivate, repression of viral genes that drive transcription must be overcome, yet it remains unclear if the mechanisms that lead to the de-repression of viral genes is conserved in epithelial cells and neurons.

**Fig 1:**
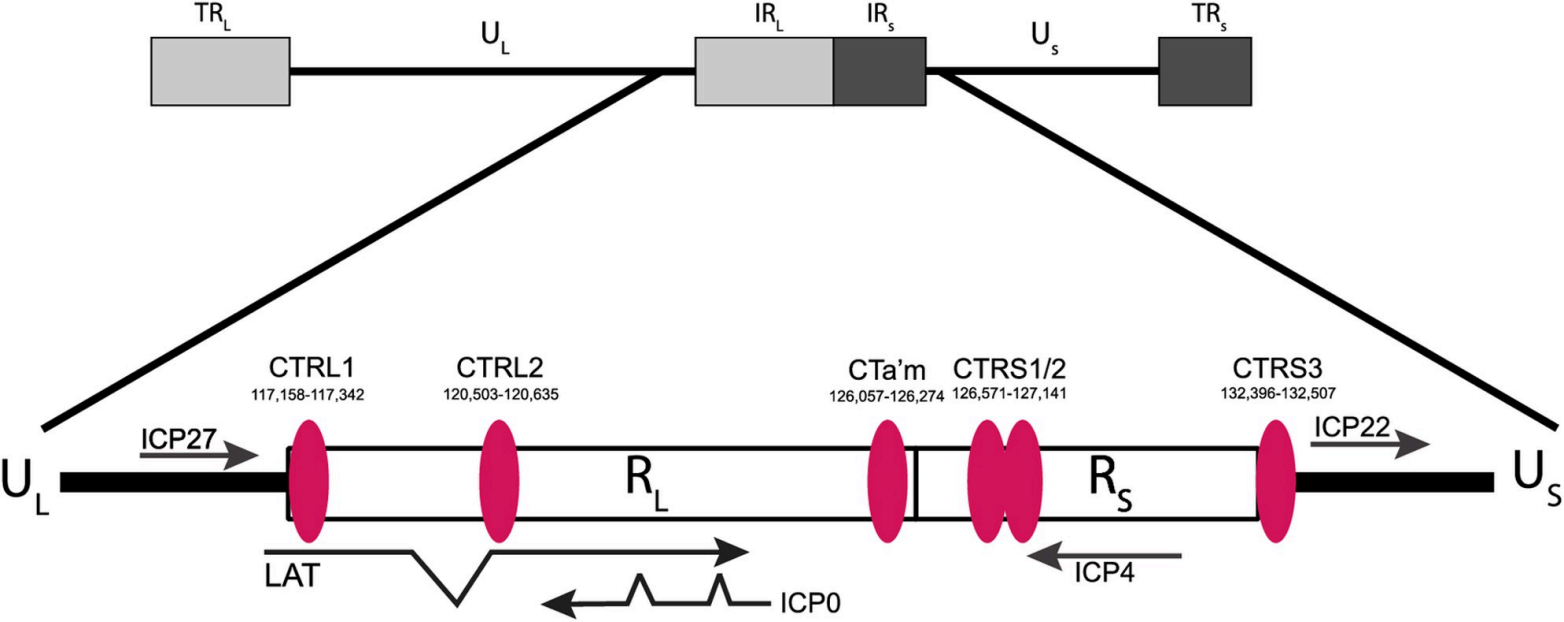
Schematic representation of previously identified CTCF insulators in the HSV-1 genome. Nucleotide positions map to previously reported CTCF sites. The CTRL2 insulator is located downstream from the 5’exon region of LAT (nt 120,503–120,635) [25]. Figure created with BioRender.com.

In sensory neurons, the latent HSV-1 genome is segregated into distinct repressive and permissive transcriptional domains that are demarked by CCCTC-binding factors, or CTCF insulators. To date, seven distinct CTCF insulators have been characterized in latent HSV-1 genomes [25–27]. The locations of the individual insulators are interesting; they flank the LAT and each of the IE genes, suggesting that individual CTCF insulators are key elements in maintaining IE gene silencing during the latent infection. In support, we showed that a specific CTCF insulator known as the CTRL2 insulator, located downstream of the LAT 5’exon region of HSV-1 (**Fig 1)**, is a key functional element in the maintenance of latency in neurons. Deletion of the core CCCTC binding site of this insulator (ΔCTRL2 recombinant) resulted in decreased heterochromatin occupancy of IE genes, increased lytic gene expression in the sensory neurons, disrupted the establishment of latency and failed to efficiently reactivate from neurons [28,29].

Mechanisms by which CTCF insulators control transcription have been well characterized in mammalian cells. They act as enhancer-blockers that prevent aberrant transcription from distal promoters or as barriers that prevent heterochromatin encroachment onto euchromatic genes and they protect the integrity of transcriptional domains through spatial organization of genomes [30–34]. However, in contrast to their silencing properties, CTCF insulators have also been implicated in regulating RNA polymerase II (RNAPII) activity, including recruitment, elongation, and transcriptional pausing [35,36]. This suggests that CTCF insulators serve as fundamental and dynamic elements in controlling transcription across diverse physiological contexts.

Given the significance of CTCF insulators in transcriptional regulation (both silencing and activation) and their abundant association with the HSV-1 genome during the latent stage of infection, we hypothesized that CTCF insulators in the HSV-1 genome also modulated transcription of adjacent viral genes during the initial lytic HSV-1 infection. In support, during our initial characterization of the ΔCTRL2 recombinant used in latency studies, we observed that this recombinant virus displayed a genome replication defect in epithelial cells [28]. This defect was not observed in neuronal cells, suggesting that the CTRL2 insulator may have a different role in the HSV-1 lytic infection. In support of this, Lang, *et*. *al* [37] reported that CTCF proteins are abundantly associated with incoming viral genomes and play a role in promoting HSV-1 genome replication. Global knockdown of CTCF protein in HSV-1 infected cells resulted in increased heterochromatin enrichment on the lytic promoters, a reduction in RNAPII occupancy on viral genomes and decreased viral transcription, suggesting that CTCF proteins promote transcription of virus by limiting silenced chromatin on the genome during the initial lytic infection [37].

To further parse out how an individual functional CTCF insulator contributes to the initial chromatinization and transcription of viral genes during the lytic infection, we leveraged our ΔCTRL2 recombinant virus in a series of experiments designed to determine the role of the CTRL2 insulator. Compared to our wild-type parent 17*Syn+*, we observed a genome replication defect and decreased IE and early (E) gene expression in the absence of the CTRL2 insulator in both fibroblasts and epithelial cells. Further, decreased IE gene expression was confirmed in the absence of protein synthesis in the ΔCTRL2 recombinant, suggesting that the defect in gene expression resulted from chromatin (in) accessibility and not decreased IE protein abundance. Chromatin immunoprecipitation (ChIP) assays showed decreased enrichment of the cellular proteins HCF-1 and Oct-1 on the IE promoters of HSV-1 at early times following infection in the absence of the CTRL2 insulator, further suggesting that chromatin accessibility was affected by the deletion of the insulator in the recombinant virus.

To further elucidate mechanisms of how the deletion of the CTRL2 insulator specifically influenced chromatin accessibility, we performed ChIP experiments to determine the enrichment of Alpha-thalassemia/mental retardation, X-linked chromatin remodeler (ATRX), and the histone variant H3.3, both of which are known for their roles in maintaining repressive histone markers on the viral genome. We found that ATRX enrichment was significantly higher on the ICP0 promoter region, and that H3.3 deposition on all IE promoters increased in the absence of the CTRL2 insulator. Finally, both H3K27me3 and H3K9me3 repressive histone marks were more enriched on the IE regions in the absence of the CTRL2 insulator in the absence of protein synthesis, strongly suggesting that the CTRL2 insulator significantly impacts the chromatin associated with the IE genes of viral genomes during the initiation of the lytic infection. Collectively, these data suggest that the CTRL2 insulator is required for 1) initial de-repression of ICP0, and 2) removal of heterochromatic marks on the IE promoters. To our knowledge these are the first data that show that a specific CTCF insulator in the HSV-1 genome (CTRL2) regulates chromatin accessibility in a lytic infection.

## Results

### Viral genome replication is attenuated in the absence of the CTRL2 insulator in fibroblasts and epithelial cells

**Fig 2:**
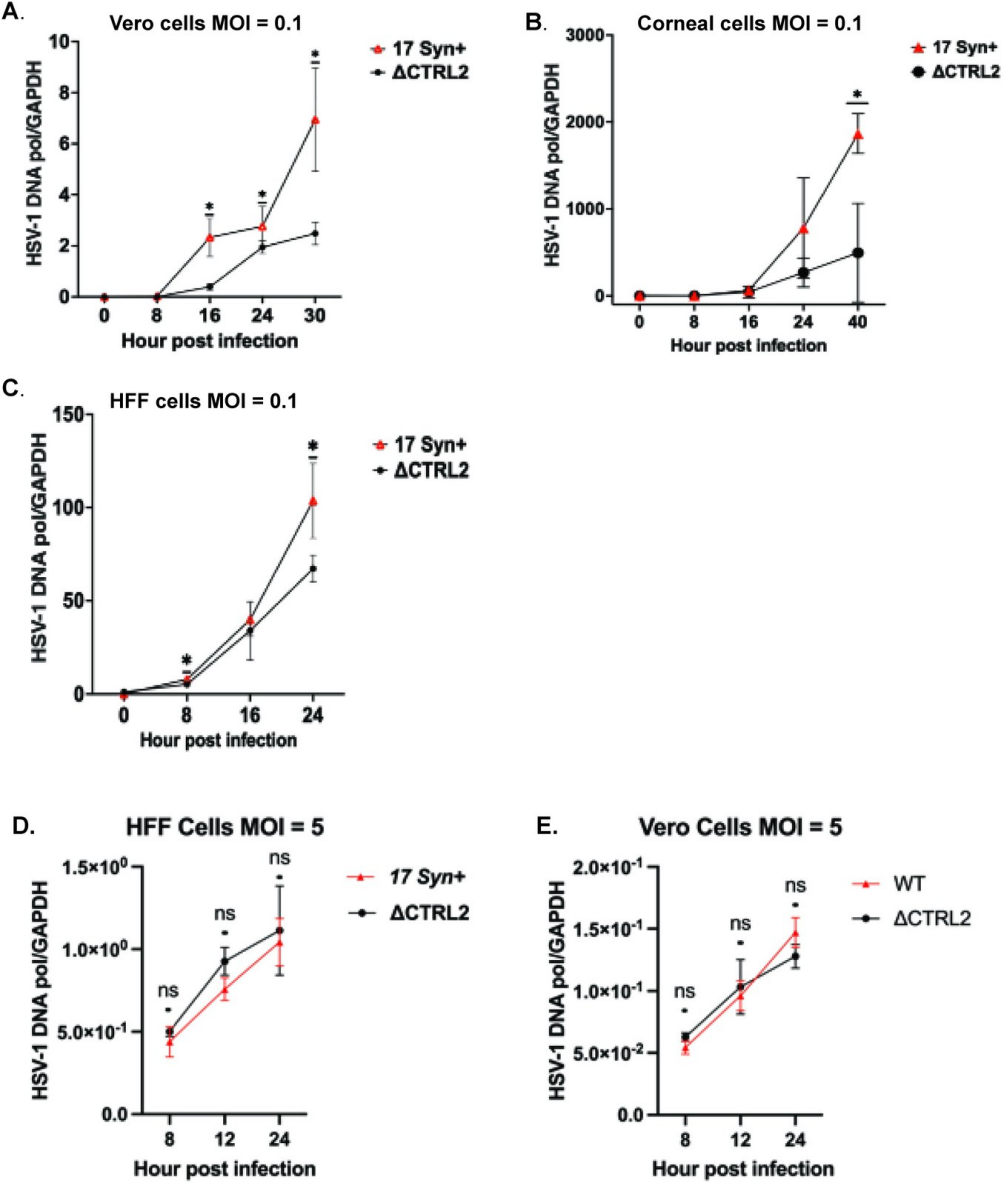
Multi-step growth curves identified defects in genome replication across cell types in the absence of the CTRL2 insulator. Confluent monolayers of Vero, HFF and corneal epithelial cells were infected with either wt 17Syn+ or ΔCTRL2 at an MOI of 0.1. Cells were harvested and DNA isolated at the specified time points in each graph. Genome replication curves were determined using qPCR for HSV-1 DNA polymerase and each value was normalized to the cellular control GAPDH. **A.** Relative HSV-1 DNA pol values obtained in Vero cells were normalized to rhesus GAPDH. **B.** Relative HSV-1 DNA pol values obtained in corneal epithelial cells were normalized to human GAPDH. **C.** Relative HSV-1 DNA pol values obtained in HFF cells were normalized to human GAPDH. **D and E.** A single step growth curve was done for HFF cells and Vero cells. Cells were infected at an MOI of 5 with either the wt or mutant viruses and DNA was harvested. qPCR was done using the viral gene HSV-1 DNA pol and normalized to total ng of nucleic acid for each sample. Each timepoint represents 3 independent experiments (n = 3). *p<0.05 by student’s t-test.

We previously reported that deletion of the CTRL2 insulator resulted in a replication defect in rabbit skin cells and 3T3 cells, but not neuro 2A cells suggesting that this specific insulator had a cell-type specific phenotype [28]. To determine whether this genome replication defect was consistent across multiple cell types including primary cell lines, we quantified viral genome replication in both single step and multi-step growth curves for both wild-type (wt) 17*Syn+* and the ΔCTRL2 recombinant virus in cells commonly used to study HSV-1 lytic genome replication, including Vero cells, normal human foreskin fibroblasts (HFF) and primary corneal epithelial cells. For the multi-step growth curves, cells were infected at an MOI of 0.1 and DNA was harvested across times ranging from 8 hours post infection (hpi) to 40 hpi. Total DNA was isolated, and purity was determined by NanoDrop One/OneC Microvolume UV-Vis Spectrophotometer. qPCR was done using primers and a probe specific for the HSV-1 DNA polymerase gene (Table 1). Relative values for HSV-1 DNA pol were normalized to the appropriate host GAPDH and plotted over time. We found significant genome replication defects (ranging from 2.5-6-fold decreases) in cells infected with the ΔCTRL2 recombinant virus relative to wt in all cell types analyzed (**Fig 2A, 2B and 2C).** These data confirmed that the CTRL2 insulator is an important element required for efficient viral genome replication during the lytic phase of HSV-1 infection under conditions that resemble natural infection. Using both Vero and HFF cells, single step growth curves were done with an MOI of 5, using time points of 8, 12 and 24 hpi. When we performed single step growth curves, we found no defect in genome replication in either cell type **(Fig 2D and 2E)**. These data suggest that the viral genome replication machinery and/or genome replication kinetics are not affected by the loss of the CTRL2 insulator or that the virus is able to overcome defects at high MOI in a single, synchronized genome replication event. Further, while a multi-step growth curve cannot directly measure chromatin dynamics on incoming viral genomes, it does suggest that there could be potential issues with chromatinization of the mutant viral genome that led to genome replication defects overall, key concepts that are further experimentally tested in this manuscript with more direct assays (ChIP).

**Table 1:** Primer/probe sequences used for ChIP, qPCR and qRT-PCR.

Primer		Sequence (5’ to 3’)
ICP0-TAATGARAT (ChIP)	Forward	CTT ATA CCC CAC GCC TTT CC
	Reverse	GGC ATG CTA ATG GGG TTC T
	Probe	CGC CCC CAA AGA ATA TCA TT
ICP4- TAATGARAT (ChIP)	Forward	ATTACCGCCGAACCGGGAAG
	Reverse	CGCATGGCATCTCATTACC
	Probe	CCCGTTCCTCGTTAGCATG
ICP0 Spliced	Forward	AGC GAG TAC CCG CCG GCC TG
	Reverse	CAG GTC TCG GTC GCA GGG AAA C
	Probe	AGC CCG CCC CGG ATG TCT GGG
ICP0 Unspliced	Forward	CTT TGG TTG CAG ACC CCT TTC TC
	Reverse	CAG GTC TCG GTC GCA GGG AAA C
	Probe	AGC CCG CCC CGG ATG TCT GGG
ICP4	Forward	CAC GGG CCG CTT CAC
	Reverse	GCG ATA GCG CGC GTA GA
	Probe	CGA CGC GAC CTC C
ICP27	Forward	GCC CGT CTC GTC CAG AAG
	Reverse	GCG CTG GTT GAG GAT CGT T
	Probe	CAG CAC CCA GAC GCC
ICP8	Forward	CTC AAA GCC GCT CTC CAC
	Reverse	ATG GAG ACA AAG CCC AAG AG
	Probe	GAG CGT ACA CGT ATC CCA GG
DNA pol	Forward	AGA GGG ACA TCC AGG ACT TTG T
	Reverse	CAG GCG CTT GTT GGT GTA C
	Probe	ACC GCC GAA CTG AGC A
LAT promoter	Forward	CAATAACAACCCCAACGGAAAGC
	Reverse	TCCACTTCCCGTCCTTCCAT
	Probe	TCCCCTCGGTTGTTCC
GAPDH	Forward	GCA CCA CCA ACT GCT TAG C
	Reverse	CCT CCA CAA TGC CGA AGT G
	Probe	CTG GCC AAG GTC ATC C

### IE gene expression is attenuated in the absence of the CTRL2 insulator

**Fig 3:**
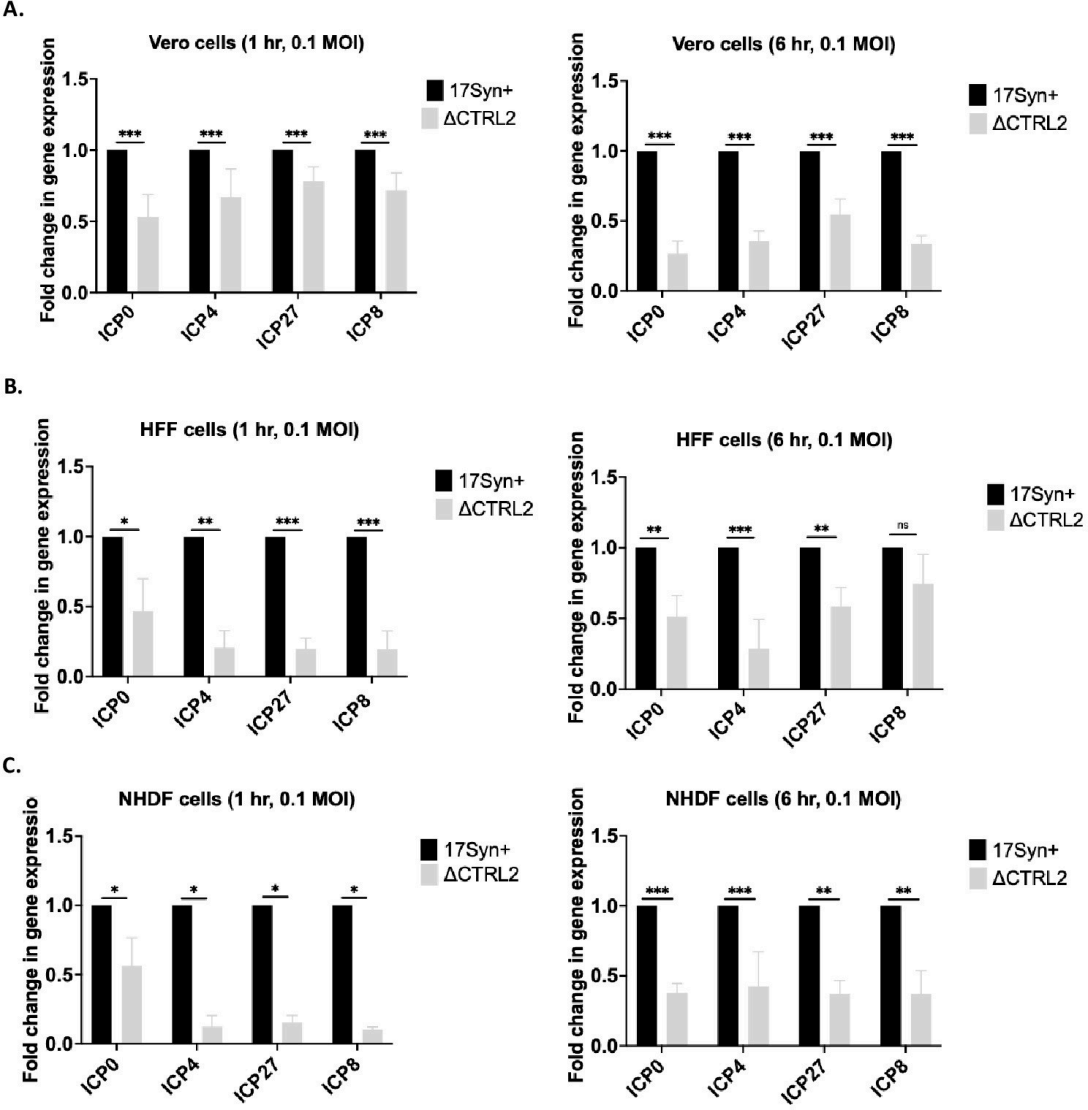
Gene expression is attenuated by 1 hpi in the absence of the CTRL2 insulator. Cells were infected at an MOI of 0.1 with either wt or ΔCTRL2 viruses and RNA extracted at 1 and 6 hpi. Gene expression for representative IE and E genes were determined in epithelial cells and fibroblasts using qRT-PCR. Gene expression was quantified using primers and probes specific for the given gene regions of HSV-1 (see Table 1). All relative values for viral genes were normalized to host GAPDH expression and were plotted as fold change in expression relative to wt virus (set to 1). Each histogram represents n = 6. **A.** Vero cell gene expression. **B.** HFF cell gene expression. **C.** NHDF cell gene expression. *p<0.05; **p<0.005; ***p<0.0005 following student’s t-test.

**Fig 4:**
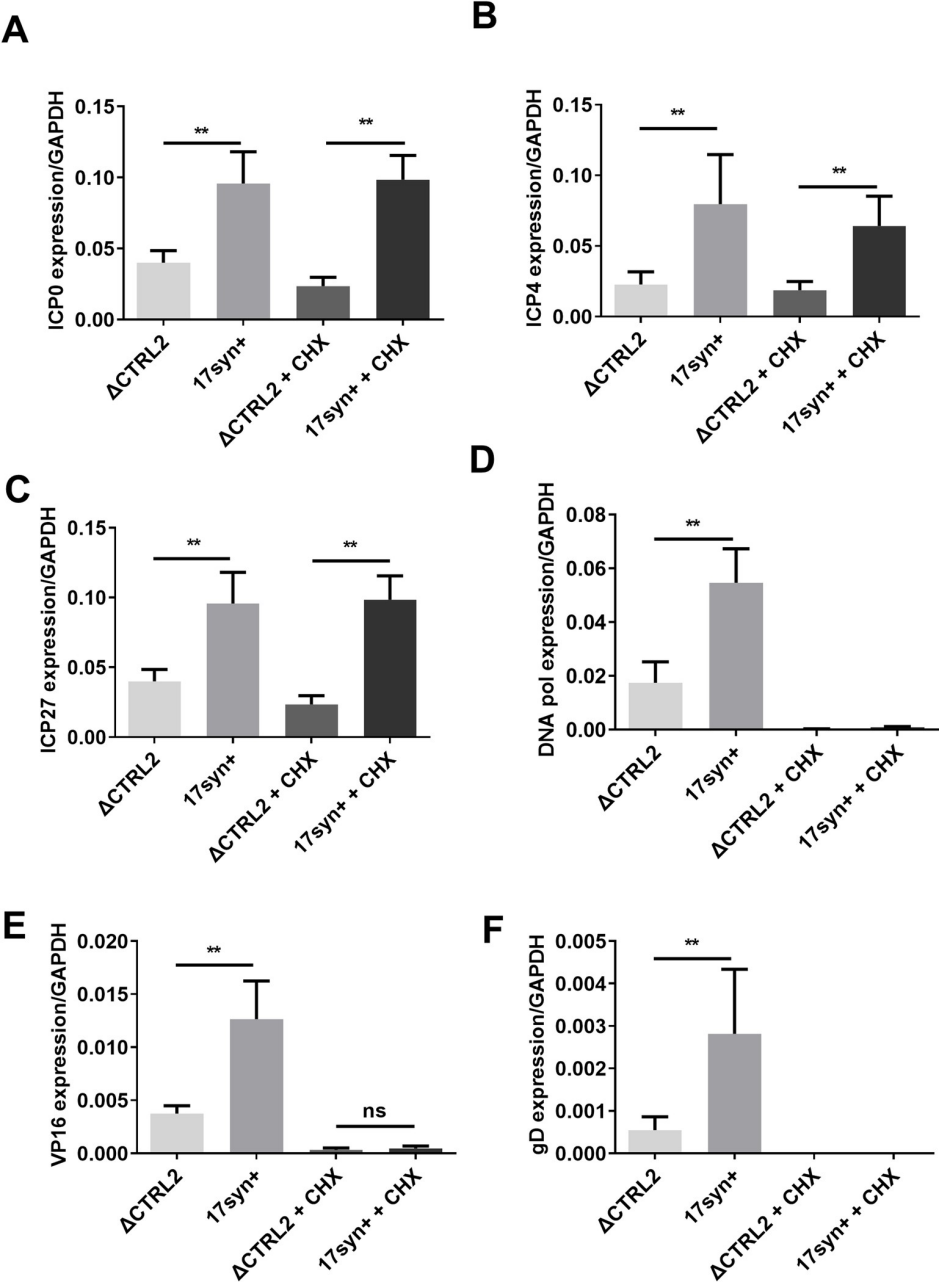
Gene expression was quantified at 6 hpi in Vero cells in the presence and absence of cyclohexamide (CHX). Cells were infected at an MOI of 0.1 with either wt or ΔCTRL2 viruses and RNA extracted at 6 hpi. Gene expression for representative IE, E and L genes were determined using qRT-PCR. All relative values for viral genes were normalized to host GAPDH expression Each histogram represents n = 6. **p<0.005 following student’s t-test.

Our first experiments told us that there was a genome replication defect in the absence of the CTRL2 insulator in HSV-1. To determine where lytic genome replication was attenuated in the ΔCTRL2 recombinant virus, we quantitated gene expression of IE and E HSV-1 genes in Vero cells as well as normal human fibroblasts (HFF and NHDF cells). Cells were infected for 1 or 6 hours at a low MOI (0.1) and total RNA was harvested following infection. qRT-PCR was used to determine relative values for the IE genes ICP0, ICP4 and ICP27 and the E gene ICP8 using primers and probes specific for those sequences (Table 1). Relative expression values for each of the HSV-1 genes were normalized to the host control GAPDH. Graphs were plotted as fold change of the normalized gene expression values relative to wt virus (set to 1). We found a significant attenuation of the expression of both IE and E genes as early as 1 hpi in all cell types analyzed compared to wt in all samples at the time-points analyzed for the ΔCTRL2 mutant (**Fig 3)**. Vero cells were then pretreated with the protein synthesis inhibitor cycloheximide (CHX) prior to infection at a concentration of 10 μg/mL for 1 h at 37°C. Cells were then infected at an MOI of 0.1 with either wt or ΔCTRL2 in the presence of CHX and gene expression was measured at 6 hpi to ensure that the genome replication defects observed were not due to attenuated protein expression in ΔCTRL2. The addition of CHX did not change the phenotype of decreased IE expression for either virus (**Fig 4),** and both E and L gene expression was inhibited (as expected due to viral protein synthesis inhibition), suggesting that genome replication defect stems from a defect in IE gene expression and chromatin accessibility of the genome may play a role in this phenotype.

### VP16 localizes to the nucleus in the absence of the CTRL2 insulator

**Fig 5:**
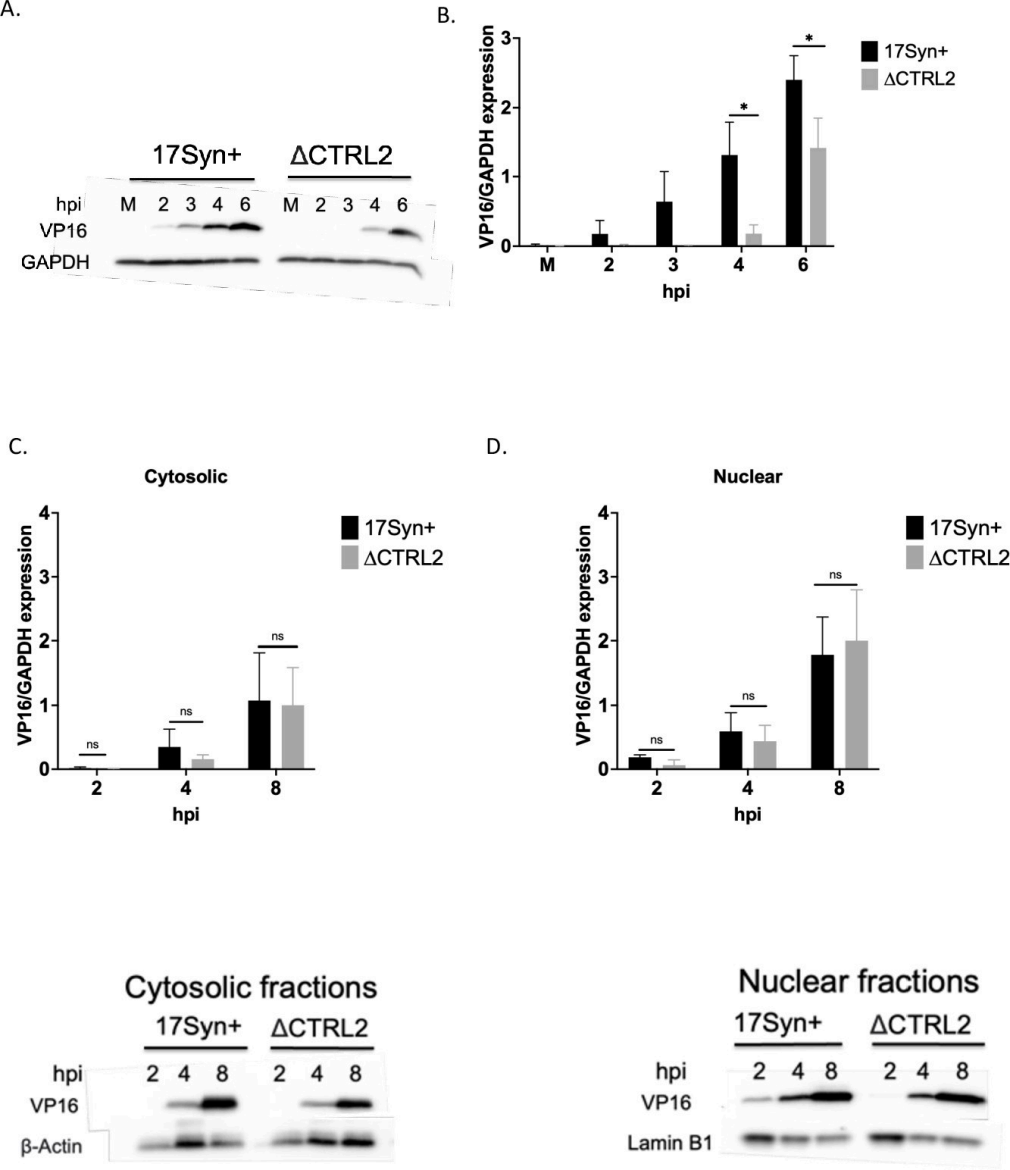
VP16 protein abundance is decreased at low MOI in the ΔCTRL2 recombinant virus. **A.** Representative western blot is shown for VP16 protein abundance. Vero cells were infected at an MOI of 1 and total cell lysates were harvested at 0 (mock = M), 2, 3, 4 or 6 hpi. **B.** Quantification of protein expression was done by ImageJ using 3 biological replicates infected with wt or ΔCTRL2 the recombinant virus at MOI of 1 and band intensities were normalized to the cellular control GAPDH. **C and D.** VP16 protein abundance in cytosolic and nuclear fractionated samples was quantitated to show at high MOI (5) no significant differences could be detected between the wt and mutant virus through 8 hpi and VP16 localized to the nucleus in the absence of the CTRL2 insulator. Representative cytocolic and nuclear fraction western blots are shown and quantification of protein abundance was done by ImageJ using 3 biological replicates and comparing the band intensities for VP16 to β-actin in the cytosolic fractions and VP16 to Lamin B in the nuclear fractions.

**Fig 6:**
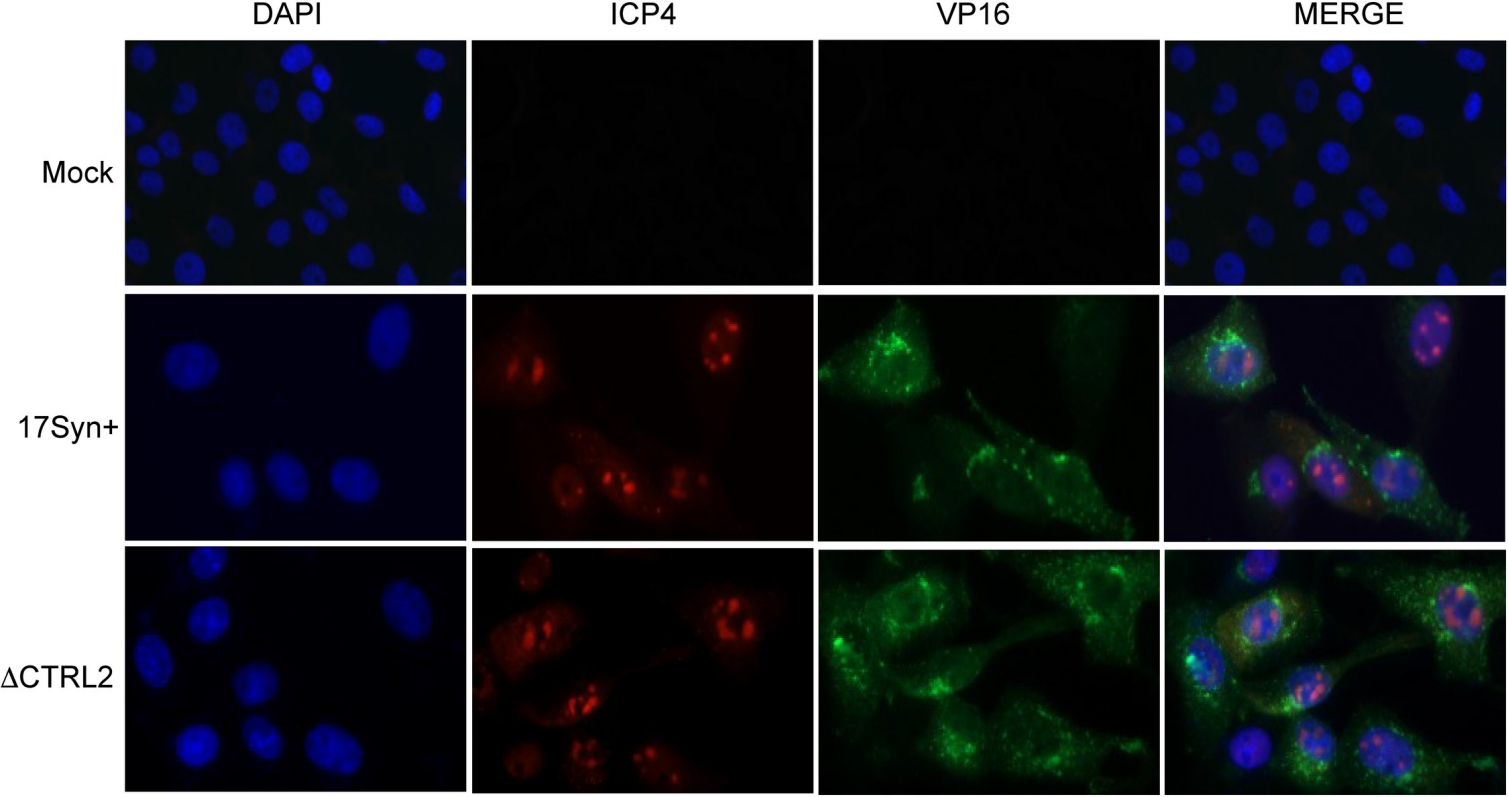
VP16 and ICP4 proteins can be found in the infected cell nucleus in both wt and ΔCTRL2 recombinant virus. Vero cells were infected with either wt or recombinant at an MOI of 5. Cells were fixed and IFF done at 5 hpi using antibodies for VP16 or ICP4. Images shown are at 20X magnification and were captured with a Zeiss Axiomager Z2 upright fluorescent microscope using ZenPro software. IFF shows that ICP4 compartmentalization and VP16 nuclear localization is indistinguishable between the two viruses. Controls for the expaeriment included mock infected cells subjected to primary and secondary antibody incubations.

Quantitation of gene expression showed that IE gene expression was significantly attenuated at 6 hpi even in the presence of CHX in ΔCTRL2. We also observed significant decreases in both E and L genes in ΔCTRL2. To confirm the decreased transcription of L genes corresponded to decreased protein expression at the lower MOI of 1, we isolated whole cell lysates from Vero cells and probed for VP16 protein accumulation by western blot at 2–6 hpi. A representative western blot is shown in **Fig 5A** for the whole cell lysates at MOI = 1. To quantitate differences in protein abundance between the mutant and wt virus, we used ImageJ to graph the intensities of VP16 relative to GAPDH in 3 biological replicates **(Fig 5B).** Here we show that VP16 protein expression is lower in ΔCTRL2 compared to wt, consistent with decreased gene expression at lower MOI shown in Fig 4. To quantitate VP16 accumulation in the nucleus of infected cells, we fractionated infected Vero cells (MOI = 5) into cytosolic and nuclear fractions and probed for VP16 expression from 2–8 hpi. Representative western blots for the cytosolic and nuclear fractions are presented in Fig 5C and 5D. To quantitate differences in protein abundance between the mutant and wt virus, we used ImageJ to graph the intensities of VP16 relative to β-actin for the cytosolic fractions and VP16 relative to Lamin B1 in the nuclear fractions in 3 biological replicates **(Fig 5C and 5D).** No quantitative differences in protein accumulation between ΔCTRL2 and wt could be detected in either the cytosolic or nuclear fractions at high MOI (**Fig 5C and 5D)**. These data are consistent with our findings that at high MOI, the genome replication defects are overcome. To visualize nuclear VP16, Vero cells were plated to 50% confluency and infected at an MOI of 5. At 5 hpi, cells were fixed with 4% PFA, incubated with first primary then secondary antibodies and then processed for image analyses. We found no obvious differences in the cellular localization of VP16 in the infected cells in the absence of the CTRL2 insulator. HSV-1, like other DNA viruses, establishes viral genome replication compartments to initiate productive infection and complete genome replication [39]. It has been reported that CTCF is required for lytic genome replication and that CTCF is localized to genome replication compartments following HSV-1 infection in fibroblasts [37]. Therefore, to determine whether there were acute differences in the localization of viral proteins associated with the formation of genome replication compartments in ΔCTRL2, we stained for the viral protein ICP4 in both mutant and wt viruses. Here, we saw no obvious differences in the ability of ICP4 to localize to the nucleus of the cells, suggesting that deletion of the CTRL2 insulator did not interfere with the nuclear localization of transactivating proteins required for genome replication or with the formation of genome replication compartments **(Fig 6)**.

### RNAPII enrichment is reduced at the promoters of IE genes in the absence of the CTRL2 insulator

**Fig 7:**
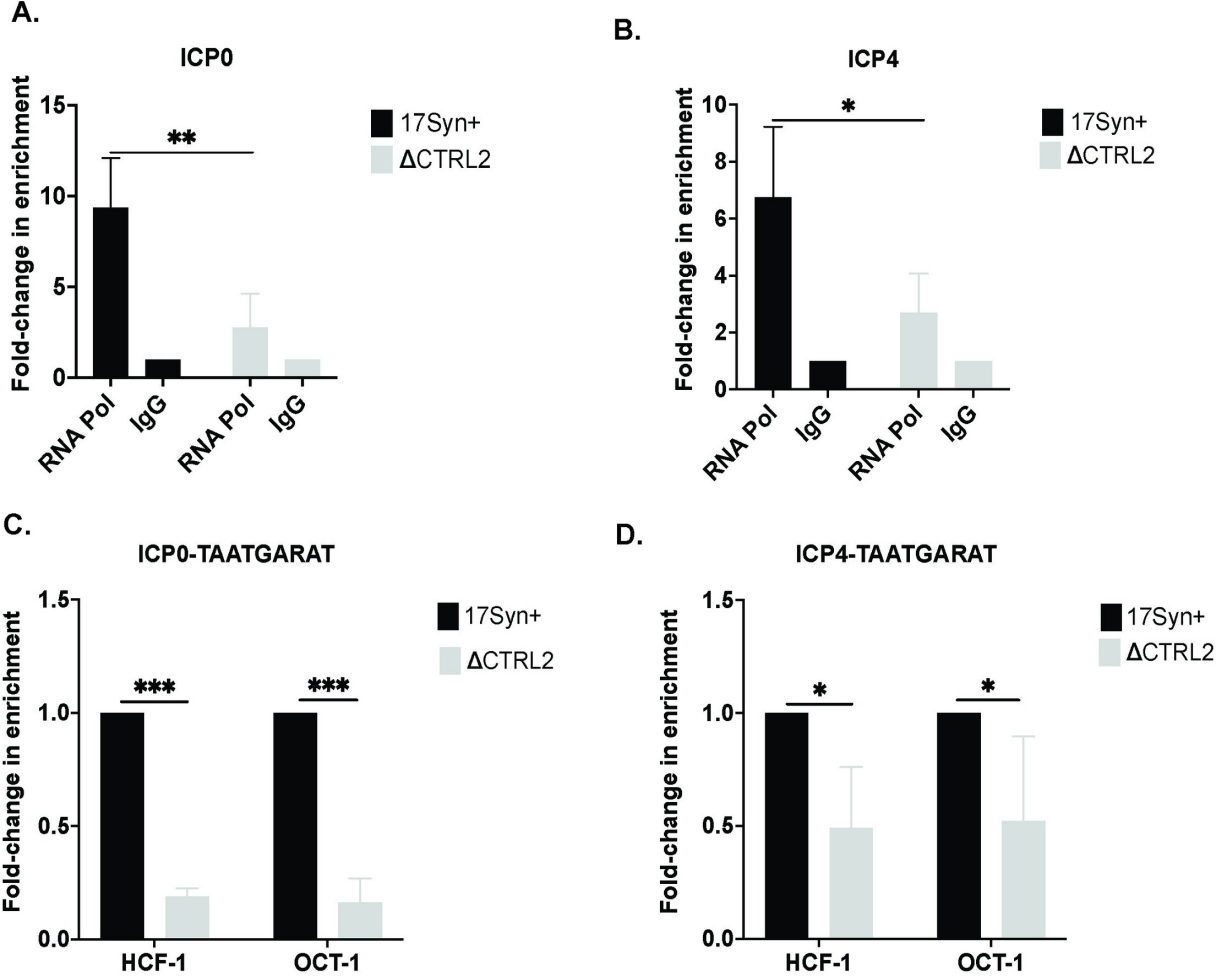
RNAPII accumulation on promoters of the IE genes was attenuated in the absence of the CTRL2 insulator. Chromatin immunoprecipitation (ChIP) assays were done using an antibody specific for RNAPII following infection. Vero cells were infected at an MOI of 1.0 with wt and ΔCTRL2 viruses (n = 5). Cells were harvested at 6 hpi and immediately crosslinked for ChIP. ChIP was performed with antibody or the IgG control. qPCR using primers listed in Table 1 were done for both antibody and IgG samples. Samples were first validated with a host control as described in the methods. The relative bound/input (B/I) ratios were normalized to IgG and are presented as fold-change relative to IgG (set to 1). **A.** Fold-change in enrichment for RNAPII for the ICP0 promoter. **B.** Fold-change in enrichment for RNAPII for the ICP4 promoter Error bars are measured for standard deviations from the mean fold change of the replicates. **C.** Accumulation of HCF-1 and Oct-1 on TAATGARAT sites of ICP0 and; **D.** ICP4 following ChIP assays (n = 5). Cells were harvested at 6 hpi ChIP was performed with each antibody or the IgG control following host control validation for HCF-1 and Oct-1. The fold-enrichments of antibody to IgG were determined for each antibody and then normalized to the fold-enrichment for wt virus (set to 1). Error bars are measured for standard deviations from the mean fold change of the replicates. *p<0.05, **p<0.005, ***p<0.0001 following students t-test.

HSV-1 utilizes host cell transcriptional mechanisms to drive gene expression, including co-opting RNA polymerase II (RNAPII) [40]. At the very earliest stages of HSV-1 infection, IE genes are the first viral genes to be expressed and are critical to promote the progression of the lytic infection. It has been previously shown that transcription of IE genes is mediated by cellular RNA polymerase II (RNAPII) and is regulated by viral proteins including VP16 as well as cellular transcription factors, transcriptional coactivators, and chromatin modulation complexes that are recruited to IE enhancer/promoters [19]. In the context of HSV-1 infection, global CTCF knockdown during the lytic infection led to a reduction of RNAPII occupancy on viral promoters [37,41], suggesting a link between RNAPII recruitment and CTCF enrichment on the viral genome. To further evaluate this in the context of the CTRL2 insulator, we infected Vero cells with either wt or the ΔCTRL2 recombinant virus and performed Chromatin Immunoprecipitation assays (ChIP) assays combined with qPCR to quantitate RNAPII enrichment on the promoters of ICP0 and ICP4 at 6 hpi. In this and subsequent ChIP assays, Vero cells were used as a representative cell type, due to their rapid doubling rate and the ability to establish robust infections using low MOI. We detected no differences between Vero or HFF cells in the genome replication and gene expression phenotypes established following infections with the mutant virus, suggesting that Vero cells were an acceptable representative cell line to perform ChIP assays. Prior to analyzing RNAPII enrichment on viral genes, we first validated the ChIP assay pulldowns using the positive control GAPDH. Because the overall pull-down efficiencies of ChIP grade antibodies differ depending on the antibody, we arbitrarily set the validation threshold for cellular control/IgG to >4 fold-change and only use ChIP assays that validate to that ratio for further analyses. Validation thresholds of >4-fold were used for all subsequent ChIP assays presented in this work as well. Following ChIP using the RNAPII antibody, we found a significant decrease in the enrichment of RNAPII on the ICP0 and ICP4 promoters in the absence of the CTRL2 insulator (**Fig 7A and 7B)**, further suggesting that the CTRL2 insulator could be required for chromatin assembly during the lytic infection [37].

### Oct-1 and HCF-1 binding is attenuated on TAATGARAT sites of IE genes in absence of the CTRL2 insulator

IE genes of HSV-1 are regulated by promoter-enhancer assemblies, of which the reiterated enhancer elements, or TAATGARAT sequences, are targeted by the viral tegument protein VP16 [42]. VP16 initiates lytic transcriptional programs through the VP16-induced complex, a complex formed from VP16 interactions with the cellular proteins Oct-1 and HCF-1, first through the association of VP16 with HCF-1 in the cytoplasm, followed by translocation of the complex to the nucleus where it then colocalizes to the host protein Oct-1 bound to the TAATGARAT sites of IE promoters. This binding ultimately drives transcription and initiates the lytic cascade [43]. Further, there is a link between HCF-1 and RNAPII enrichment on viral promoters in HSV-1 [44,45]. In our gene expression experiments we observed consistent attenuation of IE and subsequent E genes through 6 hpi in the ΔCTRL2 recombinant, suggesting that deletion of the CTRL2 insulator disrupted components of the promoter-enhancer assemblies. Unfortunately, ChIP grade antibodies for VP16 are currently unavailable, and therefore we were unable to directly quantify whether VP16 enrichment on TAATGARAT sites of IE genes was affected by the deletion of the CTRL2 insulator. Therefore, to explore whether the promoter-enhancer assemblies were disrupted, we performed ChIP to quantitate Oct-1 and HCF-1 enrichment on the TAATGARAT sites of ICP0 and ICP4 at 6 hpi. Prior to analyses of viral gene enrichments, we validated the ChIP assays using host-controls (rhesus PolR2A for Oct-1 and rhesus MMACHC for HCF-1 as Thermofisher generated TaqMan gene expression assays). ChIP experiments were done as described in the methods and our previously published manuscripts [26,27] and then combined with qPCR using primers and probes specific for either the ICP0 or ICP4 TAATGARAT sequences (Table 1). Following ChIP-qPCR, we quantitated a significant decrease in the enrichment of both HCF-1 and Oct-1 on TAATGARAT sites of both ICP4 and ICP0 in the absence of the CTRL2 insulator **(Fig 7C and 7D)**, suggesting that deletion of the CTRL2 insulator disrupts the promoter-enhancer assemblies leading to reduced host-protein enrichment to TAATGARAT sites.

### H3.3 loading on IE regions is significantly enriched in the absence of the CTRL2 insulator

**Fig 8:**
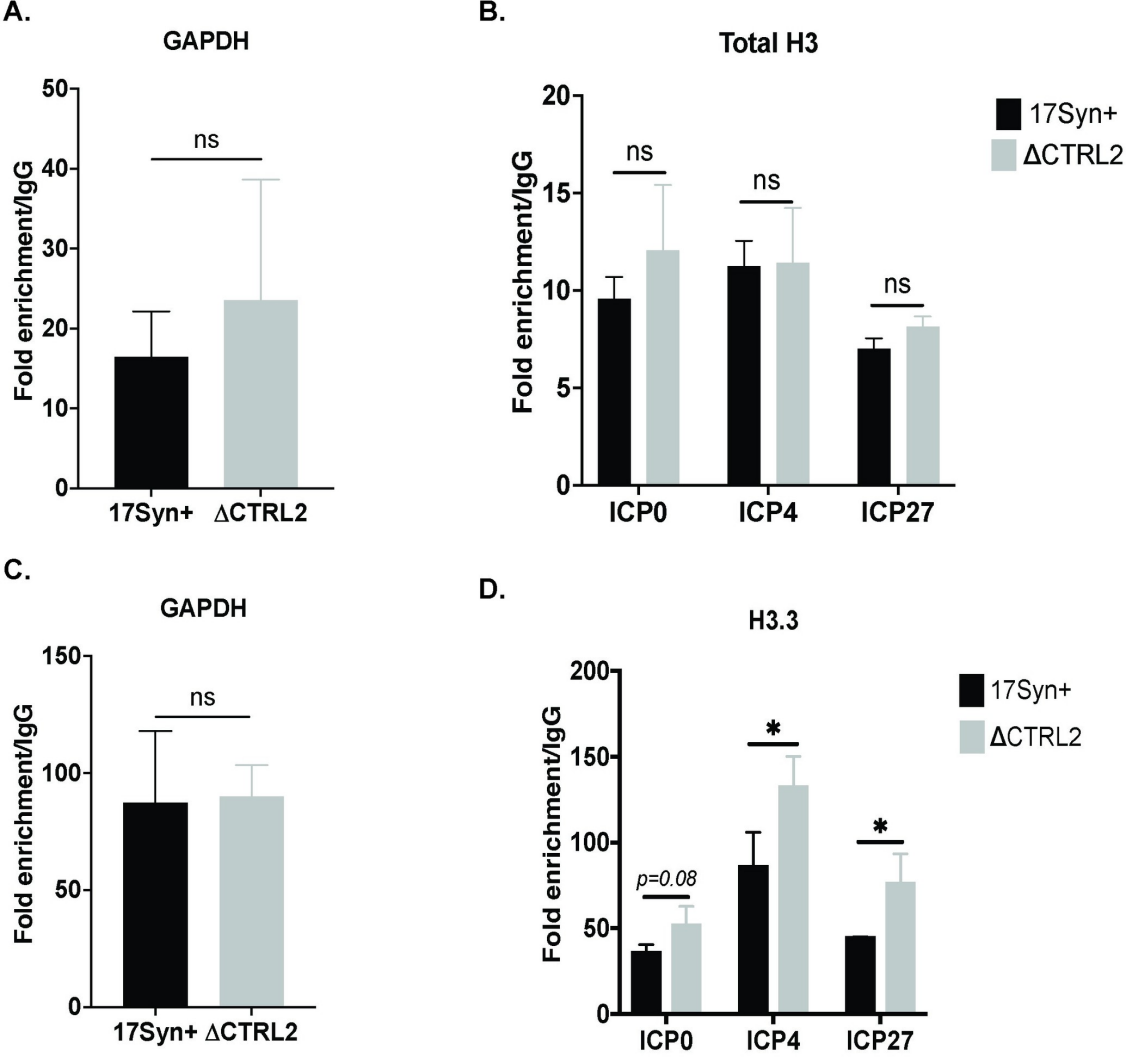
**A&C.** Fold enrichment/IgG for GAPDH were used to validate each ChIP assay with either total H3 or H3.3. There was no significant difference in total H3 or H3.3 binding on the cellular control, indicating that differences in H3.3 accumulation were limited to the viral regions analyzed and not a global enrichment. **B&D.** Total H3 enrichment and H3.3 enrichment was determined at 1 hpi by ChIP using either total H3 or the H3.3 antibodies in Vero cells infected at an MOI of 1.0 with wt and ΔCTRL2 viruses (n = 3). The fold-enrichments of H3 to IgG were determined following qPCR. Error bars are measured for standard deviations from the mean fold enrichment/IgG of the replicates. *p<0.05 following students t-tests.

The decreased enrichments of both HCF-1 and Oct-1 on the TAATGARAT sites of IE genes suggested that deletion of the CTRL2 insulator could have affected accessibility of the viral genome by altering the chromatin architecture associated with incoming viral genomes. HSV-1 genomes are rapidly loaded with histone H3 following infection, and H3 is subsequently modified with heterochromatin-associated histone modifications that maintain gene silencing. Alpha-thallasemia/mental retardation, X-linked chromatin remodeler, or ATRX, is an integral epigenetic regulator of cellular gene expression through the maintenance of silenced heterochromatin. In the context of DNA viruses, ATRX plays a role in restricting the expression of viral genes, likely by modulating heterochromatin stability during viral genome replication and transcription [46]. Together with the death domain-associated protein (DAXX), the ATRX/DAXX complex act as a histone chaperone complex specific for non-canonical histone variant 3.3 (H3.3) to maintain the heterochromatin that silences transcription from these loci [47,48]. Therefore, we reasoned that if chromatin architecture was altered in the absence of the CTRL2 insulator, we would observe differences in one or more of these components at early times following the infection of host cells. To test this, we infected Vero cells with both wt and ΔCTRL2 and performed ChIP assays at 1 hpi to determine relative fold enrichments of total H3, the histone variant H3.3 and ATRX on the IE genes of HSV-1. IE genes were selected for 2 reasons; first, these are the first genes that are activated following infection and whose expressions were altered by the deletion of CTRL2 and second, the CTRL2 deletion is directly up or downstream from ICP0, ICP4 and ICP27 promoters. The ChIP assays were validated using the cellular control GAPDH for H3 and H3.3 by determining the fold enrichment of GAPDH to IgG. In addition, we also compared GAPDH/IgG enrichment between the wt and mutant viruses to ensure that there was no significant difference in the fold-enrichments of the cellular controls. Here, three biological replicates were used for each condition and the fold-enrichments of GAPDH ranged from 15-fold to 40-fold in the individual samples. While we detected no significant differences in the fold-enrichments/IgG between the wt and mutant virus cellular controls, the sample size (n = 3) might be underpowered to detect significant differences in cellular controls between the two viruses in the case of total H3 (**Fig 8A)**. Nonetheless, following ChIP with the total H3 antibody, we found that there was no significant difference in total H3 enrichment to either the cellular control or to any of the IE genes analyzed (**Fig 8A and 8B).** However, we found significant enrichment of the histone variant H3.3 at both ICP4 and ICP27 and, while not significant, there was a trending increase in the enrichment of H3.3 on ICP0 as well in the absence of the CTRL2 insulator (**Fig 8D)**. In contrast, we detected no differences in enrichment of H3.3 on the cellular gene GAPDH in either virus following ChIP (**Fig 8C).**

**Fig 9:**
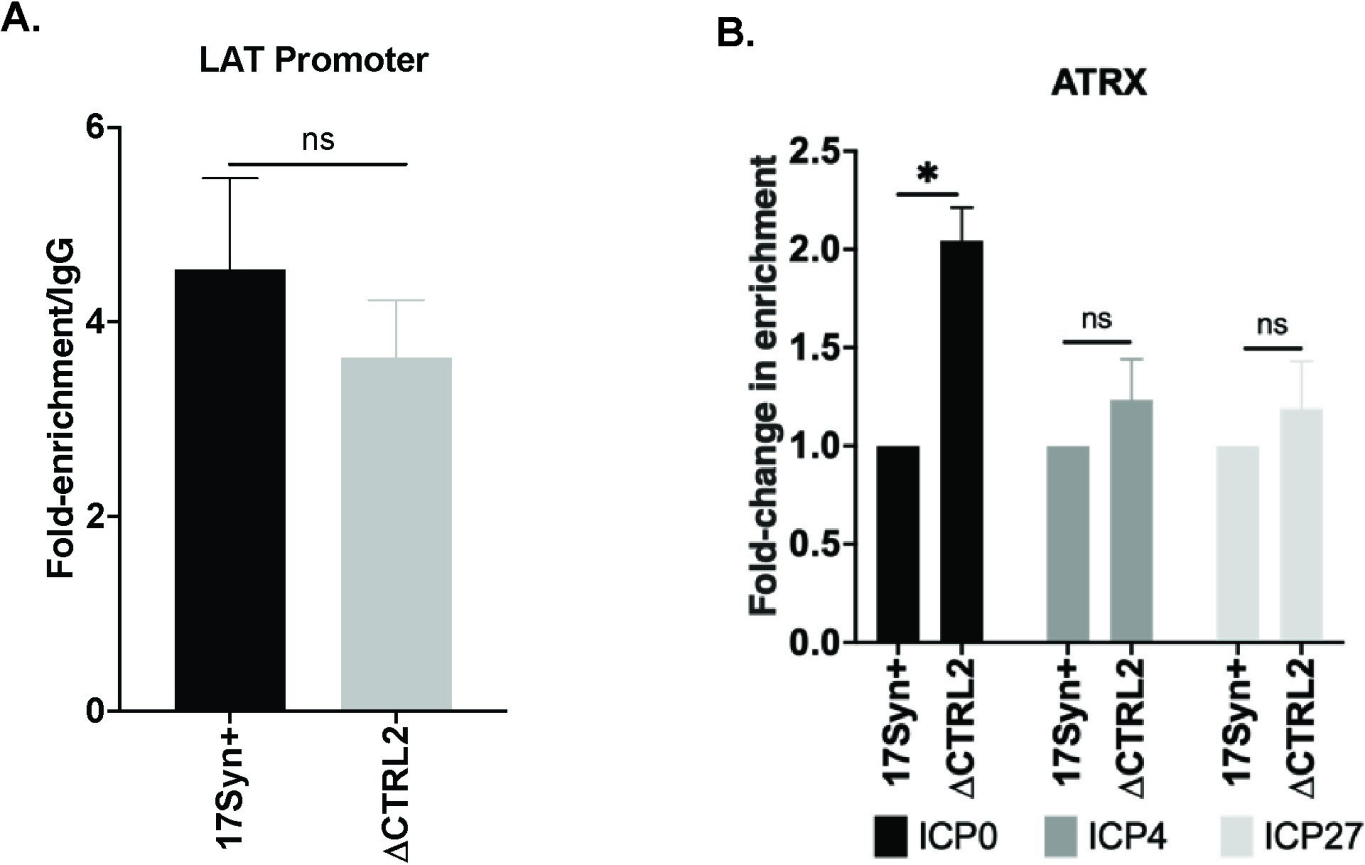
**A.** Fold enrichments/IgG for the LAT promoter were used to validate ChIP assays with the ATRX antibody. **B.** ATRX enrichment was determined at 1 hpi by ChIP in Vero cells infected at an MOI of 1.0 with wt and ΔCTRL2 viruses (n = 3). The fold-enrichments of ATRX to IgG were determined following qPCR. Relative fold-enrichments are plotted as fold-change normalized to wt virus (set to 1). Error bars are measured for standard deviations from the mean fold change in enrichment for each of the replicates. *p<0.05, **p<0.005, ***p<0.0001 following students t-test.

We next determined ATRX enrichment on the viral IE genes at 1 hpi. ATRX pulldowns were validated using the viral LAT promoter, where all enrichments remained >3-fold over IgG (**Fig 9A).** Relative enrichments of ATRX were determined using fold-enrichment of ATRX over IgG at the individual viral promoter sites analyzed and the data is shown as the fold-change in ATRX enrichment relative to the respective wt promoters (**Fig 9B).** Interestingly, comparison of ATRX enrichment on IE regions in the wt and ΔCTRL2 recombinant revealed that only ICP0 was significantly, but moderately, enriched (∼2-fold) in the absence of the CTRL2 insulator relative to wt infected cells. This was an interesting finding, as it suggests that the loss of the CTRL2 insulator site resulted in the dysregulation of the neighboring IE gene ICP0 through ATRX localization on that region of the genome. This finding is further discussed in the discussion section of the manuscript. Nonetheless, collectively these data suggest that the CTRL2 insulator of HSV-1 is required for chromatin accessibility at very early times in the lytic infection.

### H3K27me3 and H3K9me3 enrichment on IE regions of HSV-1 is significantly higher in the absence of the CTRL2 insulator

**Fig 10:**
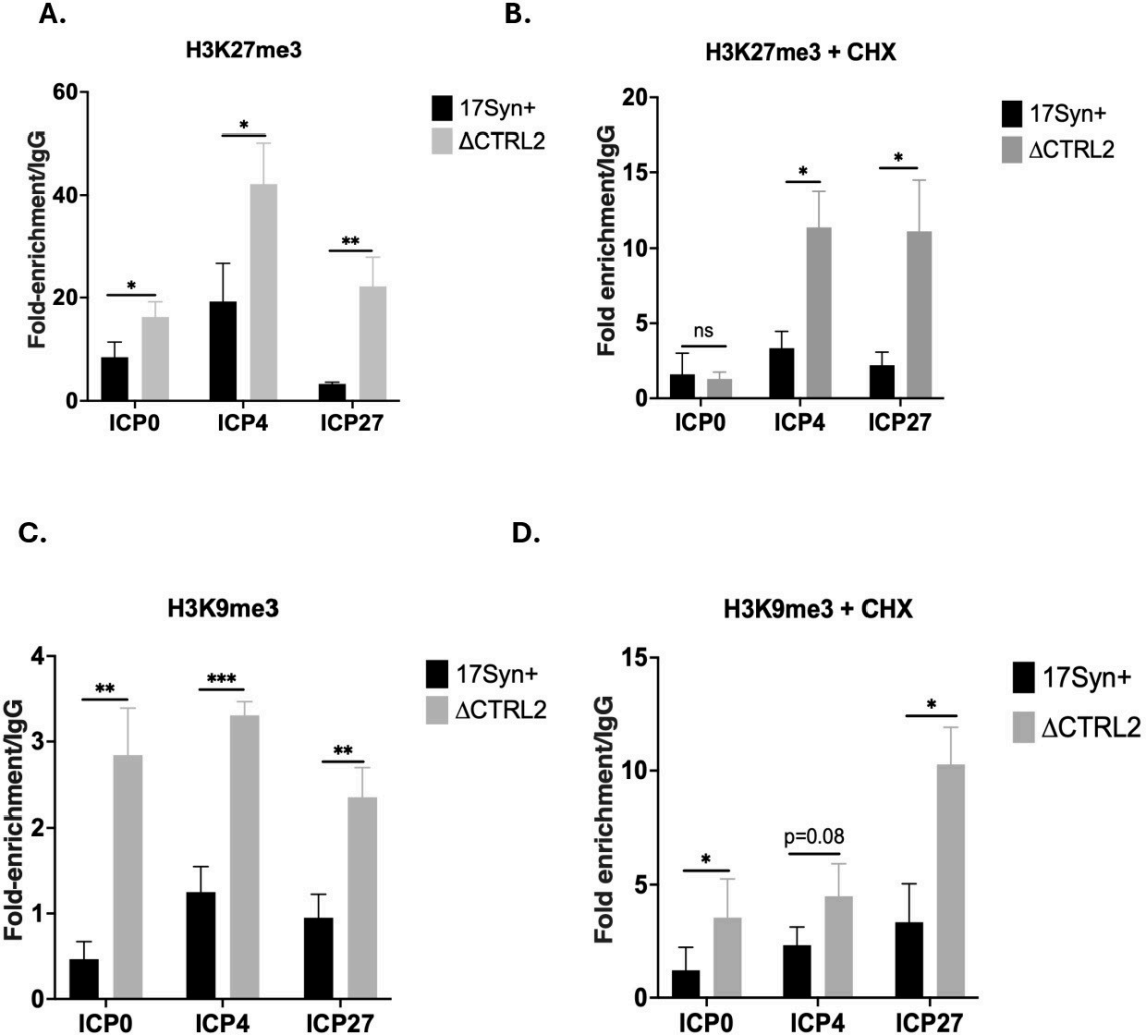
**A.** H3K27me3 enrichment was determined at 4 hpi by ChIP in Vero cells infected at an MOI of 1.0 with wt and ΔCTRL2 viruses (n = 3). **B.** H3K27me3 enrichment was determined at 4 hpi by ChIP with wt and ΔCTRL2 viruses in the presence of CHX (n = 3). **C.** H3K9me3 enrichment was determined at 4 hpi by ChIP in Vero cells infected at an MOI of 1.0 with wt and ΔCTRL2 viruses (n = 3). **D.** H3K9me3 enrichment was determined at 4 hpi by ChIP with wt and ΔCTRL2 viruses in the presence of CHX (n = 3). The fold-enrichments of H3K27me3 (or H3K9me3) compared to IgG were determined following qPCR. Error bars are measured for standard deviations from the mean fold enrichment/IgG of the replicates. *p<0.05 following students t-tests.

H3.3 deposition has been linked to the deposition and maintenance of the repressive histone markers H3K27me3 and H3K9me3. In addition, Lang, *et al* have shown that global knockdown of CTCF significantly increases the enrichment of repressive chromatin marks H3K27me3 and H3K9me3 on HSV-1 viral gene promoters following lytic infection, galvanizing a connection between CTCF on the viral genome and the balance of repressive histone marks, albeit through undefined mechanisms [37]. To determine how the CTRL2 insulator directly impacts chromatin modifications and the maintenance of H3K27me3 and/or H3K9me3 on lytic promoters, we infected Vero cells and quantified for heterochromatin enrichment at 4 hpi using ChIP-qPCR in the presence and absence of CHX to control for viral protein synthesis. ChIP assays were validated using the positive host control SAT2 prior to analyses. Here we found that deletion of the CTRL2 insulator resulted in increased enrichments of the repressive H3K27me3 and H3K9me3 marks on IE regions of HSV-1 in the absence of CHX **(Fig 10A and 10C)**. ChIP assays done in the CHX showed very similar results, with H3K27me3 enrichment significantly higher on ICP4 and ICP27 of ΔCTRL2, while H3K9me3 was significantly enriched on ICP0 and ICP27 in ΔCTRL2, and while technically not significant, trended higher on ICP4 (**Fig 10D)**. Notably, we did not detect significant enrichment of H3K27me3 on the ICP0 promoter in the presence of CHX in ΔCTRL2 compared to wt (**Fig 10B)**. These data suggest that the abundance of H3K27me3 on ICP0 could be dependent on protein synthesis. Nonetheless, collectively these experiments suggest that the CTRL2 insulator has direct and significant impacts on the nature of the viral chromatin on the IE promoters.

### ATRX depletion in HFF cells restores gene expression of the ΔCTRL2 recombinant

**Fig 11:**
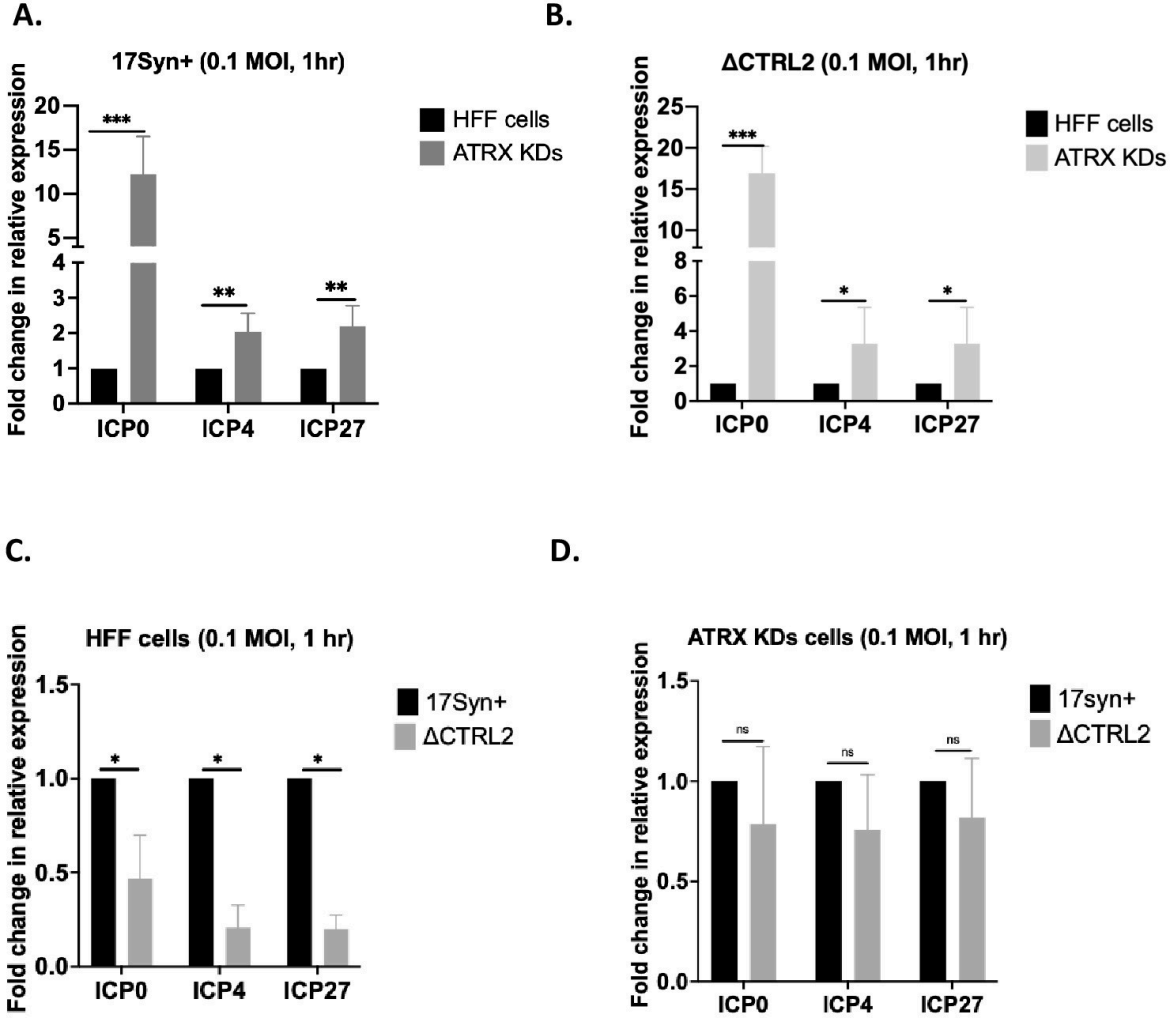
Gene expression was quantified by qRT-PCR in normal HFF cells and ATRX knock down (KD) cells. Cells were infected at an MOI of 0.1 with either wt or ΔCTRL2 viruses and RNA extracted at 1 hpi. Gene expression for the ICP0, ICP4 and ICP27 genes were determined. Gene expression was quantified using primers and probes specific for the given gene regions of HSV-1 (see Table 1). All relative values for viral genes were normalized to host GAPDH expression and were plotted as fold change in expression relative to wt virus (set to 1). Each histogram represents n = 6. **A.** 17syn+ gene expression for HFF and HFF-ATRX KD cells. **B.** ΔCTRL2 gene expression for HFF and HFF-ATRX KD cells **C and D.** Comparison of expression for both wt and mutant viruses in HFF and ATRX KD cells. *p<0.05; **p<0.005; ***p<0.0005 following student’s t-test.

ATRX restricts the expression of viral genes likely by modulating heterochromatin stability during viral genome replication and transcription [46]. Our ChIP assays suggest that the CTRL2 insulator mediates ATRX enrichment on the ICP0 region of HSV-1 as well as the deposition or removal of H3.3 on the viral genome. Therefore, we further reasoned that ATRX depletion would restore the wt phenotype in the absence of the CTRL2 insulator. To test this, we obtained ATRX Knock Down HFF cells (a gift from the Kalejta lab-UW Madison) and infected them (together with wt HFF cells) with either 17Syn+ or ΔCTRL2 recombinant viruses. Here we showed that in the absence of ATRX, gene expression for IE genes ICP0, ICP4 and ICP27 was significantly higher compared to normal HFF cells infected with 17Syn+ at 1 hpi (**Fig 11A)**, a finding consistent with previously reported data showing ATRX depletion leads to increased DNA genome replication and increased gene expression, even in the absence of ICP0 [46, 49]. Infection of ATRX depleted cells with the ΔCTRL2 recombinant virus also yielded significantly higher IE gene expression over normal HFF cells at 1 hpi (**Fig 11B)** and finally, by comparison to wt, the gene expression defect was rescued back to the wt phenotype in the absence of ATRX (**Fig 11C and 11D).** This further stratifies mechanistic role for the CTRL2 insulator in ATRX deposition to the viral genome.

### Deletion of the CTRL2 insulator does not alter CTCF enrichment in the adjacent insulator sites of HSV-1

**Fig 12:**
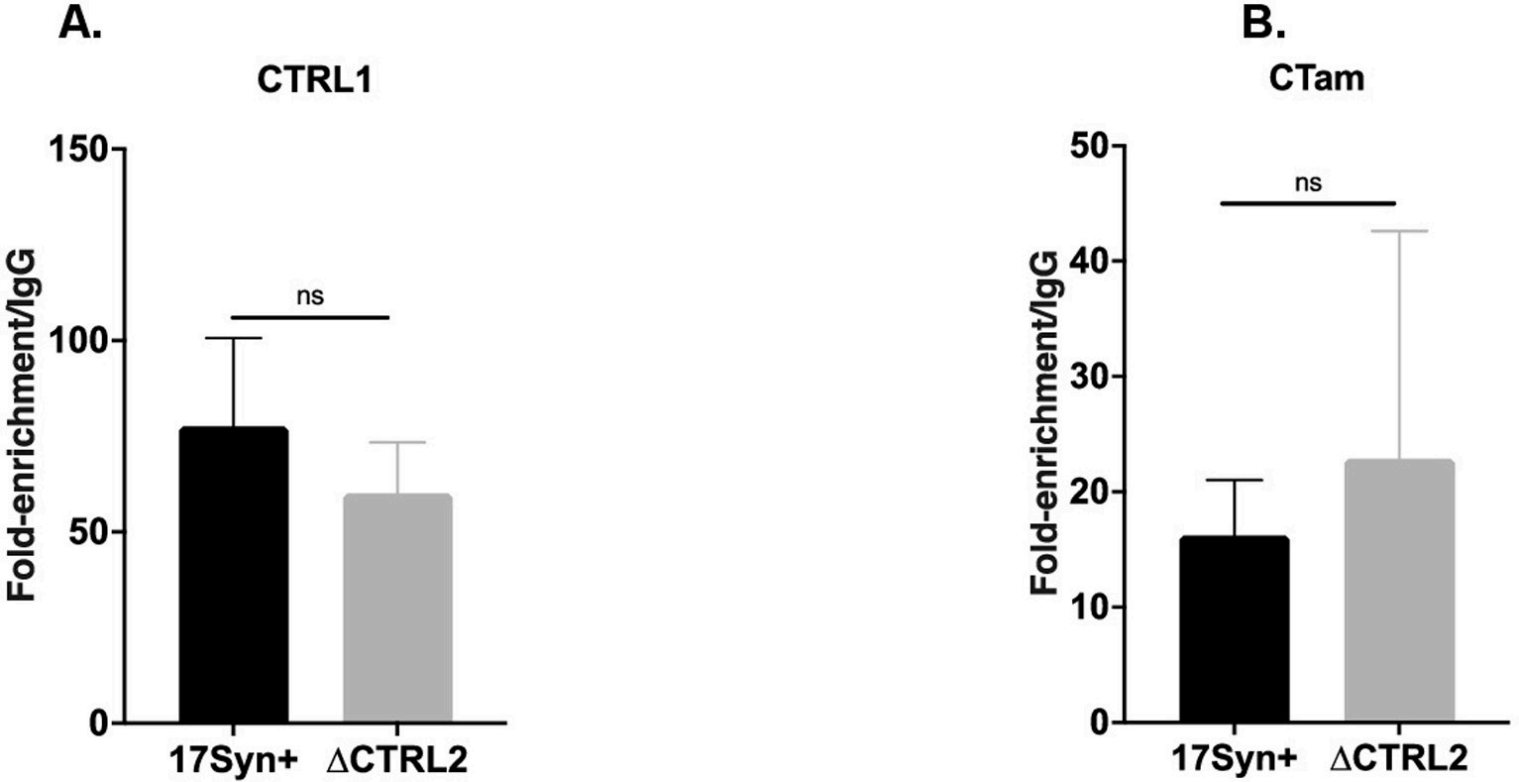
Fold enrichments/IgG for CTCF enrichment was determined at 4 hpi by ChIP in Vero cells infected at an MOI of 1.0 with wt and ΔCTRL2 viruses (n = 3). The fold-enrichments of CTCF compared to IgG were determined following qPCR using primers for sequencing flanking the CTRL1 or CTa’m insulators [38]. Error bars are measured for standard deviations from the mean fold enrichment/IgG of the replicates.

Finally, we explored the possibility that adjacent CTCF insulators located in the repeat long regions of the viral genome (Fig 1) played a role in limiting the transcription of IE genes in the absence of CTRL2. In the context cellular insulators, deletion of a CTCF binding site can redistribute CTCF binding to neighboring insulators to stabilize chromatin architecture in an ATRX-dependent manner [59,60]. The HSV-1 genome has multiple CTCF insulators that flank each of the IE genes, including two insulators adjacent to the lesion made in the deletion of the CTRL2 insulator. To determine how the deletion of the CTRL2 insulator affected CTCF enrichments at the CTCF insulator binding sites CTa’m and CTRL1 (**see Fig 1)** we infected Vero cells with either wt or ΔCTRL2 and performed ChIP using the anti-CTCF antibody followed by qPCR using unique primers corresponding to regions just outside the core CTCF binding sites. Here we found that CTCF enrichment of the adjacent insulators is unaffected in the absence of the CTRL2 insulator at either the CTRL1 and CTa’m insulators (upstream ICP27 and ICP0 respectively) (**Fig 12).** These data suggest that IE transcription is not diminished due to redistribution of CTCF proteins on other insulators.

## Discussion

CCCTC-binding factors, also known as CTCF insulators, are essential regulators of chromatin structure and gene expression in mammalian cells [32–34]. It is becoming increasingly clear that these cellular insulator elements also play vital regulatory roles in transcriptional control of all classes of herpesviruses as well [50–53]. However, much of the existing research focusing on CTCF and herpesviruses have been done in the context of viral latency but considering the role of CTCF insulators in organizing chromatin architecture, it is logical that these elements also play a role in either the initial chromatinization of virus, the de-repression of chromatin on incoming viruses so that transcription can occur- or both. Finally, while both the incoming and latent viruses are chromatinized it is unclear if the mechanisms are conserved or divergent. Understanding these mechanisms is particularly important for future drug discovery where cellular elements contributing to the de-repression of viral chromatin might be targeted to prevent reactivation.

The latent HSV-1 genome has at least 7 functional insulators that flank the IE and LAT regions of the genome and we have long speculated that these individual insulators silence transcription of viral genes during latency but maintain a genome organization that allows for the expression of those genes once reactivation is stimulated through the formation of chromatin loop structures [27,28,38]. To this point, individual CTCF insulators near the LAT region have been characterized as enhancer-blocking and/or barrier insulators while insulators around the IE genes co-localize with repressive protein complexes to maintain repressive chromatin environments of these gene regions during the latent infection in sensory neurons [26,28,54]. However, while the mechanistic roles of individual CTCF insulators have been explored in the context of neurons, little is known about their roles in transcription of the viral genome following entry into a host cell. Previous characterization of the CTRL2 insulator, located downstream of the LAT promoter/enhancer, provided evidence that deletion of this insulator disrupted chromatin architecture; the repressive H3K27me3 histone marks abundant on the wt viral genome during latency were significantly reduced in the absence of the CTRL2 insulator [28,54]. This corresponded to increased IE gene expression in neurons infected with the ΔCTRL2 recombinant. It is probable that 3D chromatin loop structures play an important role in regulation of the viral genome, at least during the latent infection. Because of this, our previous publications, as well as the data provided here, compared the ΔCTRL2 recombinant to wt virus rather than a rescuant. We routinely use wt parental viruses for our controls because, while rescue viruses are useful in proving that the genetic lesion is responsible for a change in phenotype, the process of generating the rescue results in genetic drift of the rescue from the wt virus following the transfection and multiple rounds of plaque purification. It is possible that these genetic drifts can result in significant differences in the 3D genome structures or chromatin architecture between the wt and rescue viruses. For these reasons, we propose that recombinant virus comparisons to wt virus are the more robust control for our experiments. Additionally, ΔCTRL2 has been completely sequenced and contains no additional mutations outside of the CTRL2 lesion [28,29,38]. In stark contrast to what we and others have published during latency, we found decreased lytic gene expression combined with increased enrichments of both H3K27me3 and H3K9me3 repressive marks on IE promoters in the absence of the CTRL2 insulator in lytically infected cells. These data provide some of the first evidence that CTCF insulators may utilize divergent mechanisms to control transcription of the HSV-1 genome during different stages of the HSV-1 life cycle.

In the context of HSV-1 infection, ATRX, initially identified as a transcriptional repressor and a chromatin remodeler, has been found to localize to viral promoters and limit viral gene expression following entry into the host cell [14,46,48,49]. ATRX is ultimately degraded by ICP0 in PML NBs so that viral genome replication can occur [12–14]. Our initial experiments showed a genome replication defect that precipitated from decreased IE gene expression in the absence of the CTRL2 insulator. We considered the possibility that this defect was a direct result of inefficient synthesis of ICP0 in the mutant virus, such that ATRX was not effectively degraded in PML NBs. We reasoned that if this were the case, we would not observe a gene expression defect between the wt and recombinant viruses in the presence of the protein synthesis inhibitor, cycloheximide. This was not the case, further implicating that chromatin accessibility was altered in the absence of the CTRL2 insulator. ChIP assays supported this conclusion with evidence that there was decreased enrichment of the cellular proteins HCF-1 and Oct-1 on the IE promoters of HSV-1 at early times following infection in the absence of the CTRL2 insulator. To shed light on role of the CTRL2 insulator in chromatin accessibility, we performed a series of ChIP assays to determine how deletion of this element affected loading of the histone variant H3.3. H3.3 is involved in packaging DNA into chromatin. Recent research has shown that H3.3 is incorporated into viral chromatin during HSV-1 infection, particularly at sites associated with active transcription [11,55]. This incorporation may influence the accessibility of viral DNA to transcriptional machinery and other regulatory factors, thereby impacting viral gene expression dynamics. Using GAPDH as an unchanging cellular control, we showed that H3.3 occupancy on all three IE promoters of HSV-1 was higher in the absence of the CTRL2 insulator, suggesting that the element regulated the deposition and/or removal of H3.3 on incoming viral genomes. While it might be surprising that we detected no difference in total H3, but increased levels of H3.3, it is important to point out that the total H3 antibodies used for ChIP assays detect all histone variants and cannot distinguish differences between specific variants like H3.3; therefore, detecting overall changes in the total H3 levels is likely obscured by other variants that would be pulled down with this antibody. However, it is important to note that increased H3.3 levels (in the absence of increased total H3 levels) could also suggest that existing nucleosomes are being remodeled or that H3.3 is replacing other histone variants, a possibility that was not tested in this current study.

H3.3 turnover and removal from chromatin involve several mechanisms, primarily mediated by histone chaperones (ATRX/DAXX, HIRA and ASFs), chromatin remodelers (SWI/SNF complexes), and histone-modifying enzymes that post-translationally modify of histones (e.g. acetylases and demethylases) [56–58]. Our experiments revealed that IE promoters of the ΔCTRL2 recombinant virus retained significantly higher enrichments of repressive chromatin marks H3K27me3 and H3K9me3 compared to wt at 4 hpi, even in the presence of CHX, suggesting that the recruitment of demethylases that could remove those marks may have been affected by the deletion of the CTRL2 insulator and not deficiencies in protein synthesis further downstream. Our initial experiments indicated that the genome replication defect observed in ΔCTRL2 initiated in the IE genes and therefore we did not examine chromatin accessibility of lytic genes in either the E or L kinetic classes. Nonetheless, it is possible that repression of IE gene expression could lead to increased levels of repressive chromatin across other gene classes, and additional timepoints or experiments with recombinant viruses containing alternative insulator deletions would reveal if the phenotypes observed for the CTRL2 deletion virus are unique or whether any of the HSV-1 insulator deletions would yield similar results.

We explored the possibility that the CTRL2-mediated de-repression of the viral genome was dependent on ATRX because it has been reported that ATRX restricts viral infection by altering the structure of histone H3-loaded viral chromatin that reduces viral DNA accessibility for transcription [46,49]. Utilizing an ATRX-knockdown HFF cell line, we showed that the wt gene expression phenotype was recovered in our ΔCTRL2 recombinant in the absence of ATRX, further suggesting that chromatin accessibility of HSV-1 was ATRX dependent in ΔCTRL2. Our ChIP assays showed that ATRX was significantly enriched on the ICP0 promoter of the ΔCTRL2 recombinant. These data implicate the CTRL2 insulator in modulating ATRX loading or recruitment onto incoming viral genomes. Deletion of CTRL2 insulator could affect the loading or recruitment of ATRX onto the ICP0 promoter through alterations in chromatin structures that lead to changes in chromatin accessibility. Other possibilities include disruption of protein-protein interactions facilitated by CTCF that affect the formation of protein complexes involved in the recruitment or stabilization of ATRX, altered transcription factor binding mediated by CTCF or changes in histone modifications. The latter is supported by recent reports showing that while ATRX had no effect on the initial formation of viral heterochromatin assembly ATRX was required for the maintenance of viral heterochromatin between 4–8 hpi during challenges to chromatin stability [46,49].

**Fig 13:**
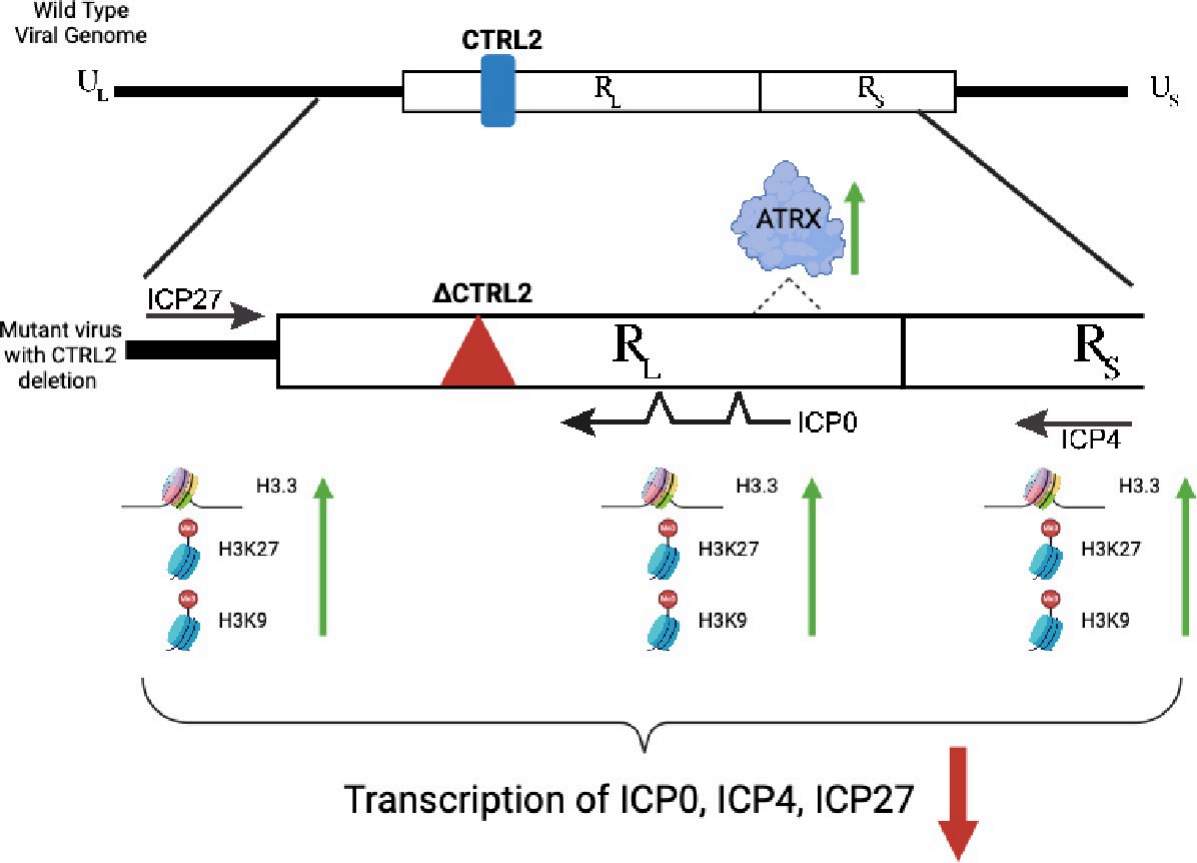
Summary model for the consequences that deletion of the CTRL2 insulator has on chromatin accessibility and chromatin associated with IE promoters during the initial lytic infection of HSV-1. Figure created with BioRender.com.

Both CTCF and ATRX have been implicated in the regulation of repetitive DNA sequences, such as repetitive elements within the HSV-1 genome. Changes in chromatin structures that play a role in regulation of these sequences can have profound effects on genomic stability and function. Here we have provided direct evidence that a link between CTCF insulator function and ATRX deposition/maintenance exists in the context of the HSV-1 IE gene ICP0. In the absence of the CTRL2 insulator, we see significantly higher ATRX associated with ICP0, suggesting that the CTRL2 insulator could act to restrict ATRX recruitment or access to DNA binding/association. Further, this link involves maintaining chromatin structure and function in intact viral genomes with respect to adjacent IE genes ICP4 and ICP27. Overall, both ATRX and CTCF insulators are important for maintaining proper chromatin structure and function, and we have shown that a deletion of a specific CTCF insulator site in the HSV-1 genome has significant consequences for transcription of HSV-1 through chromatin architecture (**Fig 13)**. These data presented suggest that the individual HSV-1 encoded CTRL2 insulator has consequences for transcription of HSV-1 by supporting that an individual CTCF insulator (CTRL2) can have significant impacts on the character of the viral chromatin associated with IE genes.

## Methods

### Cells and viruses

Vero cells (ATCC CCL-81), Human Foreskin Fibroblasts (HFF) (ATCC SCRC-1041) and human primary corneal epithelial cells (ATCC PCS-700-010) were cultured in Dulbecco’s modified Eagle’s medium (DMEM; Corning #10–017) supplemented with 10% fetal bovine serum (FBS; Corning #35-011-CV) and 1% antibiotic-antimycotic solution (Gibco #15240062) in a humidified atmosphere with 5% CO_2_ at 37°C. NHDF cells (PCS-201-012) were cultured in 15% FBS and 1% antibiotic-antimycotic solution. Cells were expanded at regular intervals when the cells were at ∼60% confluency. ATRX Knock Down HFF cells were a generous gift from the Kalejta lab (UW-Madison). ATRX KD HFF cells were supplemented with puromycin (0.5–1μg/ml) to maintain the knockdown as the integrated pLKO vector had puromycin resistance. The full characterization of these cells has been submitted for publication by the Kalejta lab [61]. Original stocks of the wild-type HSV-1 strain, 17*Syn+* (GenBank accession number NC_001806), and the recombinant ΔCTRL2 viruses were originally obtained from the Bloom Lab (University of Florida). For the ΔCTRL2 recombinant virus, 135-bp (nt 120,500–120,635) of CTRL2 insulator was deleted from the 17*Syn+* parental wild-type (wt). The recombinant virus was verified by sequence analyses, as previously described [28, 62]. Both virus stocks were propagated on Vero cells using a 0.01 MOI. Cultures were supplemented with Eagle’s minimal essential medium with 1% FBS and 1% antibiotic-antimycotic solution at 37°C for 3–4 days and viruses were harvested by centrifugation followed by two freeze thaw cycles. The final supernatants were aliquoted and stored in -80°C for further use. To determine viral titers, Vero cells were infected in triplicate with 10-fold serial dilutions in DMEM (1% FBS and 1% antibiotic-antimycotic solution) for 72 hrs, plaques were stained by crystal violet and counted. For infections prior to downstream experiments, cells were seeded and grown in 6-well plates to confluency, unless otherwise noted. Monolayers of cells were inoculated with either 17*Syn*+ or the ΔCTRL2 recombinant in DMEM with 1% FBS and 1% antibiotic-antimycotic solution. Plates were rocked at 4°C for 1 h to allow for virus adsorption, followed by 30 min incubation at 37°C, 5% CO_2_. The virus containing media was removed and replaced with fresh DMEM with 1% FBS and 1% antibiotic-antimycotic solution and plates were incubated for the remainder of the indicated time point for cell harvesting. In the cycloheximide (CHX) experiments, cells were pretreated with CHX for 1 h at 37°C at a concentration of 10 μg/mL and then for the duration of the experiment. This concentration of CHX has been previously described to be sufficient for blocking protein synthesis [63].

### Quantitative PCR for genome copies

Cells were plated at 150,000 cells per well on 6-well plates and infected at an MOI of 0.01 with HSV-1 17*Syn*+ or ΔCTRL2. Cells were harvested at the times indicated in figures and DNA was extracted using the phenol-chloroform extraction method. Genome copies were determined by quantifying relative copies of HSV-1 DNA polymerase (primer and probe sequences in Table 1) with TaqMan Fast Universal PCR Master mix (2X), no AmpErase UNG (Applied Biosystem #4352042) on an Agilent AriaMx real-time PCR machine. All relative values for HSV-1 DNA pol were normalized to host GAPDH. PCR cycling parameters were used according to the manufacturer’s recommendations.

### Quantitative reverse transcription-PCR analysis

For gene expression assays, infection was performed as described above and cells were harvested into 500 μL of TRIzol reagent (Ambion #15596026) at indicated time points. RNA was extracted according to the manufacturer’s protocol. Briefly, samples were mixed with chloroform for phase separation and precipitated in isopropanol and 75% ethanol. qRT-PCR was performed using either a SuperScript III Platinum One-step qRT-PCR kit (Invitrogen #11732088) or SuperScript III Platinum SYBR Green One-Step qRT-PCR Kit (Invitrogen 11736–059). Reactions were set up according to the manufacturer’s protocol. Primers and probes used are listed or referenced in Table 1. qRT-PCR was performed using the following thermal cycling parameters: 50°C for 15 min, 95°C for 5 min, then 40 cycles of 95°C for 15 sec and 60°C for 30 sec. Viral gene transcripts were normalized to glyceraldehyde-3-phosphate dehydrogenase (GAPDH; ThermoFisher TaqMan gene expression assay Hs03929097_gl) or rhesus GAPDH (ThermoFisher TaqMan gene expression assay Rh02621745_g1).

### Western blotting

Vero cells were seeded in T25 flasks and infected after 24 hrs with either 17*Syn*+ or ΔCTRL2 at an MOI of 3. Cells were harvested after 30 min, 1 hr, 2 hr and 4 hr post-infection and lysed in 2X lysis buffer (456 μL H2O, 120 μL 10% SDS and 24 μL **β-**ME) with protease inhibitors followed by 10 min incubation at 95°C. Samples were vortexed and spun down briefly and incubated again 10 min incubation at 95°C with 5X sample loading buffer. Samples were briefly centrifuged and loaded on a 10% SDS page followed by overnight wet western blotting transfer at 20V. The blots were developed by using a 1:1000 dilution of anti-VP16 primary antibody (Cat No. ab4808) and 1:5000 dilution of secondary antibody HRP goat anti-rabbit (Invitrogen, Ref No. 656120). For tubulin (Cat. No. 05–661) a 1:1000 dilution was used as loading control with 1:5000 dilution of secondary antibody HRP goat anti-mouse (Invitrogen, Ref No. A24500). The blot was developed with BioRad Clarity western ECL substrate. The experiment was performed a minimum three times and images were quantified with ImageJ. Student’s t-test were performed for statistical analysis. For nuclear fractionation experiments, Vero cells were seeded in T25 flasks and infected with either 17*Syn*+ or ΔCTRL2 at an MOI of 5. Cells were harvested after 2 hr, 4 hr and 8 hr post-infection and washed with 5 mL ice cold PBS with inhibitors. For cytosolic fractions the cells were lysed in 1X hypotonic buffer with detergent as per the kit (Active Motif Nuclear extract Kit, Cat No. 40010) protocol to release the nuclei. The cytosolic faction were collected in a separate tube after the centrifugation at 14,000 x g at 4°C. Cell pellets were further lysed in complete lysis buffer (Lysis buffer + 10mM DTT + Protease inhibitor Cocktail) for 30 min at 4°C and vortex for 30 sec. The nuclear fractions were collected after the centrifugation at 14,000 x g at 4°C and stored in -80°C until further use. For SDS PAGE samples were thawed on ice vortexed and spun down briefly and incubated 10 min at 95°C with 5X sample loading buffer. Samples were briefly centrifuged and loaded on a 10% SDS page followed by overnight wet western blotting transfer at 20V.

### Immunofluorescence

Vero cells were grown on 4-well chamber slides to ∼50% confluency (Lab-Tek). Cells were infected at an MOI of 5 in 1% DMEM with either 17*Syn*+ or ΔCTRL2 viruses. Following infection, cells were incubated at 4°C for 30 minutes, then transferred to 37°C with 5% CO2 for an additional 30 m. After 30 m incubation, media was removed and replaced with fresh 1% DMEM. At 5 hpi, media was removed, and cells were fixed with 4% PFA for 10 m. Cells were washed with 1X phosphate-buffered saline (PBS [pH 7.4]; Gibco) and then incubated at room temperature for 5 minutes in 0.25% Triton X-100 in PBS. Blocking was done with 1% BSA in PBS for 30 minutes at 37°C. ICP4 (1:1000 dilutions, Abcam, Cat. No. ab6514) or VP16 (1:1000 dilutions, Abcam, Cat. No. 4808) primary antibody incubations were performed for 1 h at room temperature in 1% BSA in PBS, followed by three washes with PBS for 10 minutes each. Secondary antibody anti-mouse Alexa Fluor 594 (Life technologies, Ref. No. A21203) and anti-rabbit Alexa Fluor 488 (Invitrogen, Ref. No. A21206) incubations were performed for 30 m at at room temperature, followed by three washes in PBS for 10 minutes each. Slides were mounted with Prolong Gold with DAPI (Life Technologies). Images were captured with a Zeiss Axiomager Z2 upright fluorescent microscope using ZenPro software.

### Chromatin immunoprecipitation (ChIP) assays

ChIP assays were performed for Total H3, H3.3, RNA Polymerase II, CTCF, ATRX, OCT-1 and HCF-1 using their respective specific antibodies as follows: Anti-H3 (EMD Millipore, Cat. No. 04–928), Anti-H3.3 (Abcam, Cat. No. ab176840), Anti-RNAPII (abcam Cat. No. Ab264350), Anti-CTCF (EMD Millipore, Cat. No. 07–729), Anti-ATRX (Cell signaling, Ref. No. 14820S), Anti-Oct-1 (Cell signaling, Ref. No. 8157S), and Anti-HCF1 (Cell Signaling, Cat No. 69690). Each ChIP assay performed in minimum of three biological replicates. The cells were rapidly homogenized, and the chromatin was cross-linked in 1% formaldehyde (Sigma-Aldrich). The cross-linked cell lysates were sonicated to shear the chromatin into fragments between 300 and 800 bp. Fragment size following sonication was confirmed by agarose gel electrophoresis using a 1.5% gel, as previously described [26]. The sheared chromatin was precleared with protein A-agarose/salmon sperm DNA 50% slurry (EMD Millipore, cat no. 16–157) prior to antibody incubation. An aliquot representing 1/3 of the total sample volume was removed as a sample input (I). The remaining sample was incubated in a cold room overnight with shaking with 3–5 μg of each specific antibody per 1 mL of the sample or IgG (EMD Millipore, Cat. No. PP64B) as a nonspecific antibody binding control. The chromatin-antibody complexes were collected with salmon sperm DNA protein A-agarose beads and eluted to represent the bound (B) or IgG fraction. Bound, IgG and input fractions were treated with 5 M NaCl, RNase A, and proteinase K, and the DNA was purified using a QIAquick PCR purification kit (Qiagen), as previously described [26,27]. All CHIP assays were validated with a positive cellular host control relative to IgG, as described by the antibody manufacturer documents. Because the overall pull-down efficiencies of ChIP grade antibodies differ depending on the antibody, we arbitrarily set the validation threshold for cellular control/IgG to >4 fold-change and only use ChIP assays that validate to that ratio for further analyses. It should be noted that ChIP assays were performed in Vero cells as a representative cell line, so cellular controls were rhesus homologs to the human positive control genes. All qPCR experiments were performed using TaqMan Fast Universal PCR Master mix (2X), no AmpErase UNG (Applied Biosystem #4352042) on an Agilent AriaMx real-time PCR machine using primer and probe sequences listed in Table 1. Reaction was set up according to the PCR kit’s protocol using the following thermal cycling parameters: 95°C for 2 min, then 40 cycles of 95°C for 10 sec and 60°C for 30 sec. A standard curve was generated using 10-fold serial dilutions of purified viral genome for each plate. Threshold values used for PCR analyses were set within the linear range of PCR target amplification based on the standard curve generated from each primer-probe set used.

### Statistical analysis

Data are presented as mean +/- standard deviation. N represents the number of biological replicates as independent samples. Statistical analyses were performed using GraphPad Prism, version 10.0.2. The *p* values are indicated on the graphs where a significant change was determined using unpaired two-tailed Student’s *t-*test (* denotes *p* <0.05, ** *p*<0.005, *** *p*<0.0005, ns = not significant).
